# Identification and characterization of *GmMYB118* responses to drought and salt stress

**DOI:** 10.1186/s12870-018-1551-7

**Published:** 2018-12-03

**Authors:** Yong-Tao Du, Meng-Jie Zhao, Chang-Tao Wang, Yuan Gao, Yan-Xia Wang, Yong-Wei Liu, Ming Chen, Jun Chen, Yong-Bin Zhou, Zhao-Shi Xu, You-Zhi Ma

**Affiliations:** 1 Institute of Crop Sciences, Chinese Academy of Agricultural Sciences (CAAS)/National Key Facility for Crop Gene Resources and Genetic Improvement, Key Laboratory of Biology and Genetic Improvement of Triticeae Crops, Ministry of Agriculture, Beijing, 100081 China; 2 Beijing Advanced Innovation Center for Food Nutrition and Human Health/Beijing Key Lab of Plant Resource Research and Development, Beijing Technology and Business University, Beijing, 100048 China; 3 Shijiazhuang Academy of Agricultural and Forestry Sciences, Research Center of Wheat Engineering Technology of Hebei, Shijiazhuang, 050041 Hebei China; 4 Institute of Genetics and Physiology, Hebei Academy of Agriculture and Forestry Sciences/Plant Genetic Engineering Center of Hebei Province, Shijiazhuang, 050051 Hebei China

**Keywords:** MYB transcription factor, Genome-wide analysis, Drought tolerance, Salt tolerance, CRISPR, Soybean

## Abstract

**Background:**

Abiotic stress severely influences plant growth and development. MYB transcription factors (TFs), which compose one of the largest TF families, play an important role in abiotic stress responses.

**Result:**

We identified 139 soybean MYB-related genes; these genes were divided into six groups based on their conserved domain and were distributed among 20 chromosomes (Chrs). Quantitative real-time PCR (qRT-PCR) indicated that *GmMYB118* highly responsive to drought, salt and high temperature stress; thus, this gene was selected for further analysis. Subcellular localization revealed that the GmMYB118 protein located in the nucleus. Ectopic expression (EX) of *GmMYB118* increased tolerance to drought and salt stress and regulated the expression of several stress-associated genes in transgenic *Arabidopsis* plants. Similarly, *GmMYB118*-overexpressing (OE) soybean plants generated via *Agrobacterium rhizogenes* (*A. rhizogenes*)-mediated transformation of the hairy roots showed improved drought and salt tolerance. Furthermore, compared with the control (CK) plants, the clustered, regularly interspaced, short palindromic repeat (CRISPR)-transformed plants exhibited reduced drought and salt tolerance. The contents of proline and chlorophyll in the OE plants were significantly greater than those in the CK plants, whose contents were greater than those in the CRISPR plants under drought and salt stress conditions. In contrast, the reactive oxygen species (ROS) and malondialdehyde (MDA) contents were significantly lower in the OE plants than in the CK plants, whose contents were lower than those in the CRISPR plants under stress conditions.

**Conclusions:**

These results indicated that *GmMYB118* could improve tolerance to drought and salt stress by promoting expression of stress-associated genes and regulating osmotic and oxidizing substances to maintain cell homeostasis.

**Electronic supplementary material:**

The online version of this article (10.1186/s12870-018-1551-7) contains supplementary material, which is available to authorized users.

## Background

Drought, salt and temperature stresses severely affect plant growth and agricultural production, threatening the survival of plants. Under stressful conditions, transcriptomic changes were the earliest responses in plants [1]. Gene expression analyses in plants have revealed that stress-responsive genes can be divided into two categories: effector genes and regulatory genes [2]. The products of regulatory genes, which include membrane-localized receptors, calcium sensors, kinases and transcription factors (TFs), participate in further signal transduction regulation and gene expression [1]. TFs regulate gene expression by specifically binding to the *cis*-acting elements of downstream genes to influence many important cellular processes, such as signal transduction, morphogenesis and environmental stress responses [3, 4].

Based on the characteristics of their DNA-binding domain (DBD), TFs were divided into different families, such as bZIP, MYB, NAC, ERF, WRKY and AP2 families [5–9]. The MYB TFs, which represent the largest family in plants, can be divided into different subfamilies depending on the number of adjacent repeats within the MYB domain. Each repeat forms a helix-turn-helix structure of approximately 53 amino acids [10]. MYB-like proteins with one repeat were considered MYB-related (containing a single or a partial MYB repeat), those with two were regarded as R2R3-type MYBs (2R-MYB), those with three were regarded as R1R2R3-type MYBs (3R-MYBs), and those with four repeats were regarded as 4R-MYBs [5, 9, 11–15].

The majority of MYB TFs, especially R2R3-MYBs, play important roles in response to abiotic stresses [6, 16–19]. For example, Chen identified 30 MYB genes that respond to multiple abiotic stresses in peanut [19]. *TaMYB80* improved tolerance to high temperature and drought in wheat [6]. *TaMYB56-B* enhanced tolerance to freezing and salt stresses in transgenic *Arabidopsis* [16]. Compared to R2R3-MYB TFs, the MYB-related genes were mainly characterized for their role in processes, such as the control of cellular morphogenesis, flavonoid biosynthesis, hypocotyl elongation and circadian rhythm [20–24]. *AtWER* was an early regulator of epidermal cell fate in the root and hypocotyl [21]. *Ammixta* participated in the transcriptional control of epidermal cell shape [22]. Yi et al. reported that an R1 MYB transcription factor, *GmMYB176*, regulates *GmCHS8* expression and isoflavonoid synthesis in soybean [25]. However, there were few reports that the MYB-related gene involved in abiotic stresses [12, 26]. It is important to make clear whether more MYB-related genes participate in abiotic stresses.

Soybean (*Glycine max*) is widely cultivated and is one of the most important cash crops because of its high protein and oil content. However, its growth and grain yield are severely affected by drought and salt stresses. In some crops, different MYB TFs were characterized by their support of specific roles in response to water deficit and salt stress [6, 13, 17, 19]. Despite the whole genome of soybean being sequenced years ago [27], few studies have investigated the MYB-related TFs in this species. In this study, we provided a list of MYB-related family members based on soybean genome sequencing. Further investigation revealed that a MYB-related gene, *GmMYB118*, was significantly regulated by salt and drought treatment, and overexpression of *GmMYB118* improved tolerance to drought and salt in both *Arabidopsis* and soybean. In contrast, the transformed plants of *GmMYB118* via the clustered, regularly interspaced, short palindromic repeat (CRISPR) system exhibited reduced drought and salt tolerance. Our study provides a foundation for understanding the functions of the *GmMYB118* gene in abiotic stress responses.

## Results

### Identification and chromosomal distribution of soybean MYB-related genes

**Fig. 1:**
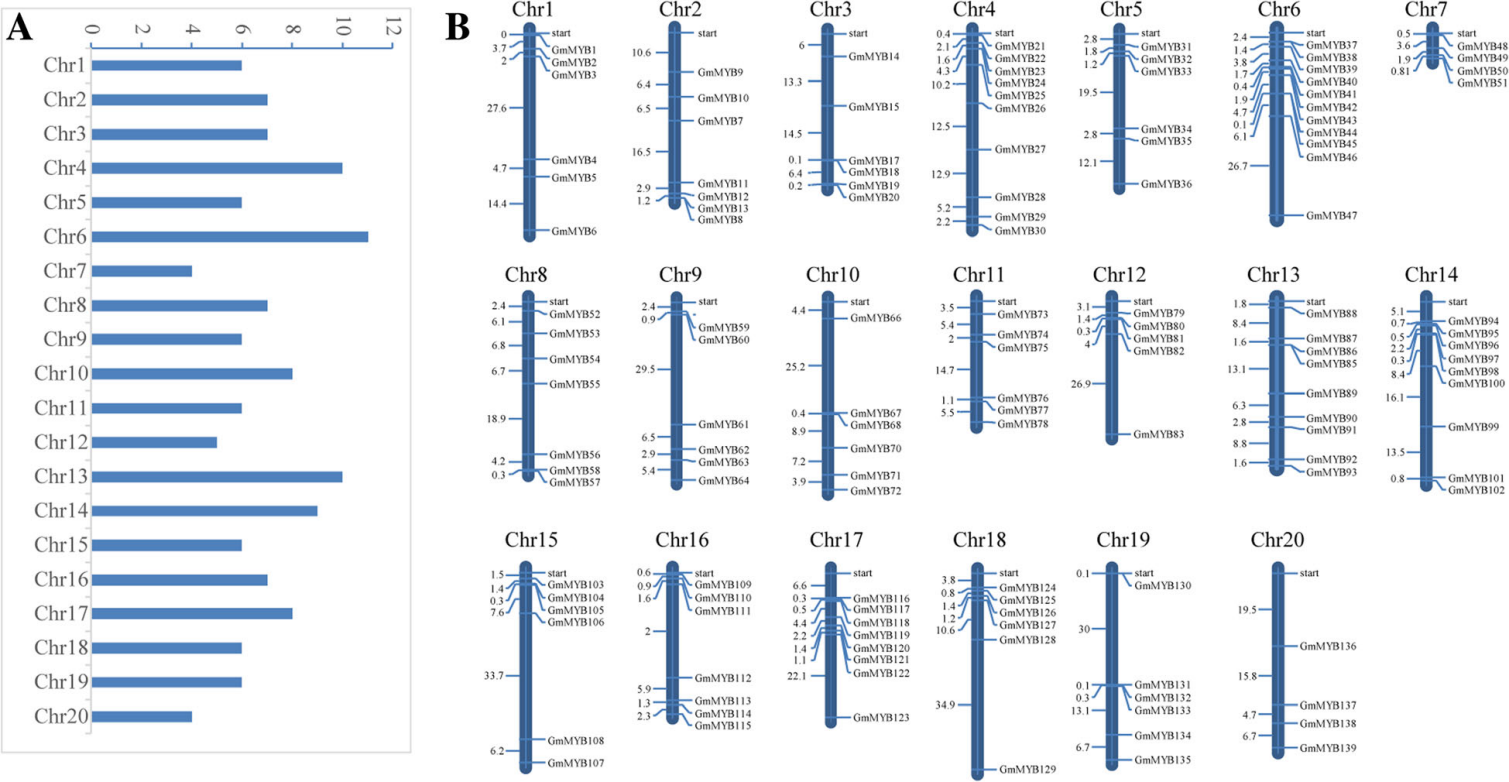
Chromosomal distribution of 139 MYB-related genes in soybean. We identified 139 MYB-related genes in soybean by researching several databases such as Phytozome, TFDB, Pfam, SMART and ScanProsite. The members of MYB-related genes were distributed on different Chr (numbers 1–20) (**a**). The physical location of each member was shown in Figure (**b**). The deep blue bars represent the Chrs, and the Chr numbers were shown on the top of the bars. The length of the bar was not represented the size of the Chr. The numbers on the left side of the bars show the distances in megabases (Mb) between neighboring genes

The species of MYB-related TF genes were various in different species (Table 1). To analyze the entire MYB-related family in soybean, we queried several databases such as Phytozome, TFDB, Pfam, SMART and ScanProsite [10]. Previous work revealed 127 MYB-related TF genes in soybean [28]; and these genes were searched against the above websites. After deleting redundant sequences and screening typical MYB-related domains, we identified 139 genes in soybean. All the MYB-related genes located on twenty chromosomes (Chrs) by using MapInspect software. Chrs 4 and 6 of soybean contain many MYB-related genes—approximately 14.8%, while fewer numbers of MYB-related genes located on Chrs 7 and 20. Chrs 5 to 9 presented a relatively uniform distribution (Fig. 1a). As shown in Fig. 1b, the MYB-related genes tended to be distributed on both arms of Chrs 9, 10, 11, 12, 15, 16, 17 and 18. On the other Chrs, the MYB-related genes were evenly distributed (Chrs 7, 8 and 13) or were abundantly distributed at either end.

**Table 1:** Numbers of MYB-related TFs in different species

Species	Number	Reference
*Arabidopsis thaliana*	68	Du et al, 2013 [28]
*Arachis hypogaea*	20	Chen et al, 2014 [19]
*Oryza sativa*	70	Dubos et al, 2010 [14]
*Zea mays*	72	Du et al, 2013 [28]
*Glycine max*	127	Du et al, 2013 [28]

### Phylogenetic tree analysis with amino acid sequence of 139 MYB-related genes

**Fig. 2:**
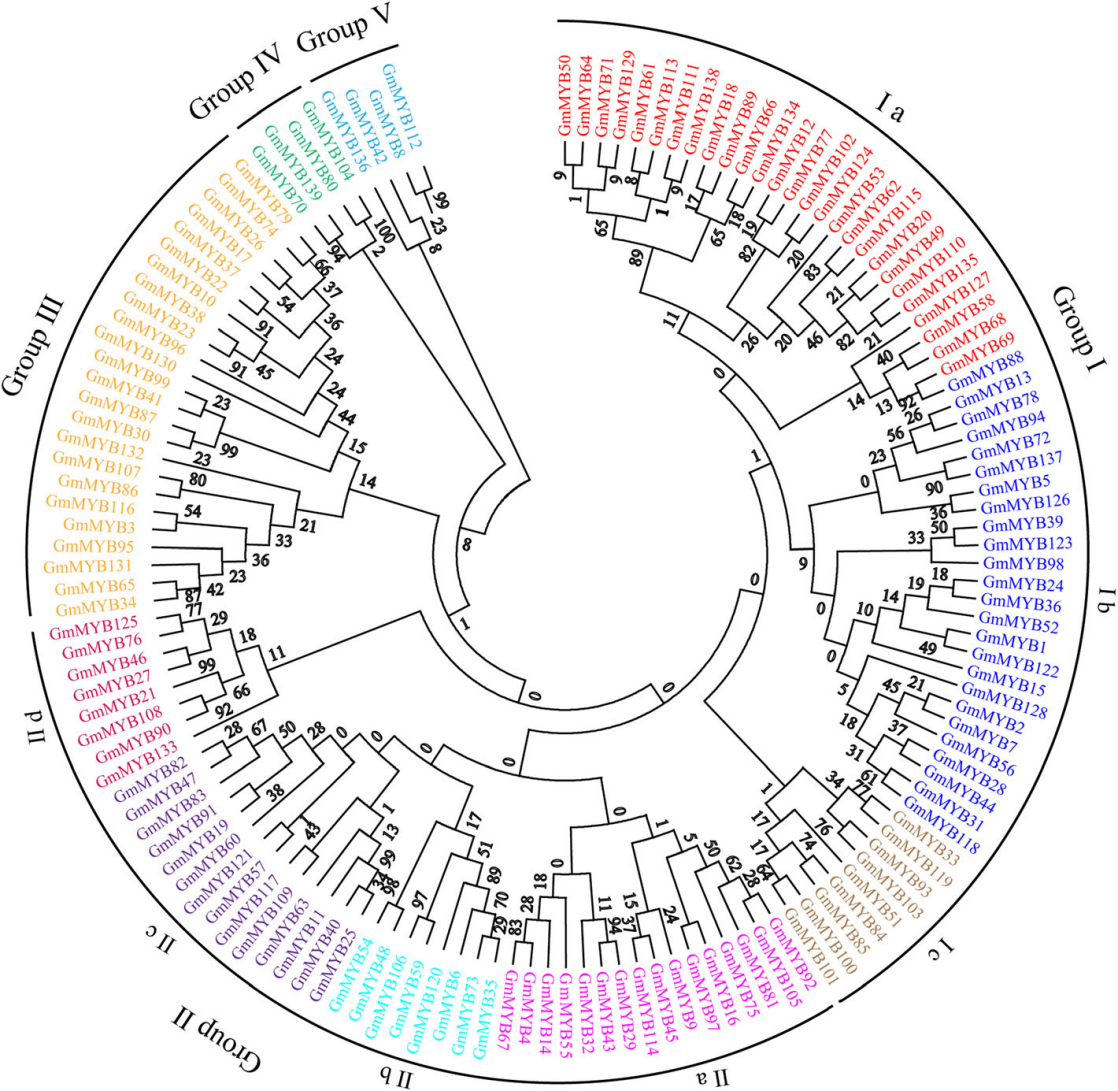
Phylogenetic tree of the MYB-related TFs subfamily in soybean. Amino acid sequences were aligned via ClustalX and were manually corrected. The phylogenetic tree was constructed with MEGA 6 in conjunction with the NJ method. The same color represents the same group

Alignment of the amino acid sequences was used to construct a phylogenetic tree by MEGA 6 via the neighbor-joining (NJ) method. As shown in Fig. 2, the phylogenetic tree was divided into 5 groups (I-V). The sequence SHAQK(Y/F) F was highly conserved in group I. Group II shared a consistent DLKDKW sequence. For other groups, although these MYB proteins have no conserved domain, they have conserved amino acid sites. The high bootstrap values for the node supported that the other members of 139 MYB proteins were clustered in three groups (III, IV and V), respectively.

### Screening candidate genes for further analysis

**Fig. 3:**
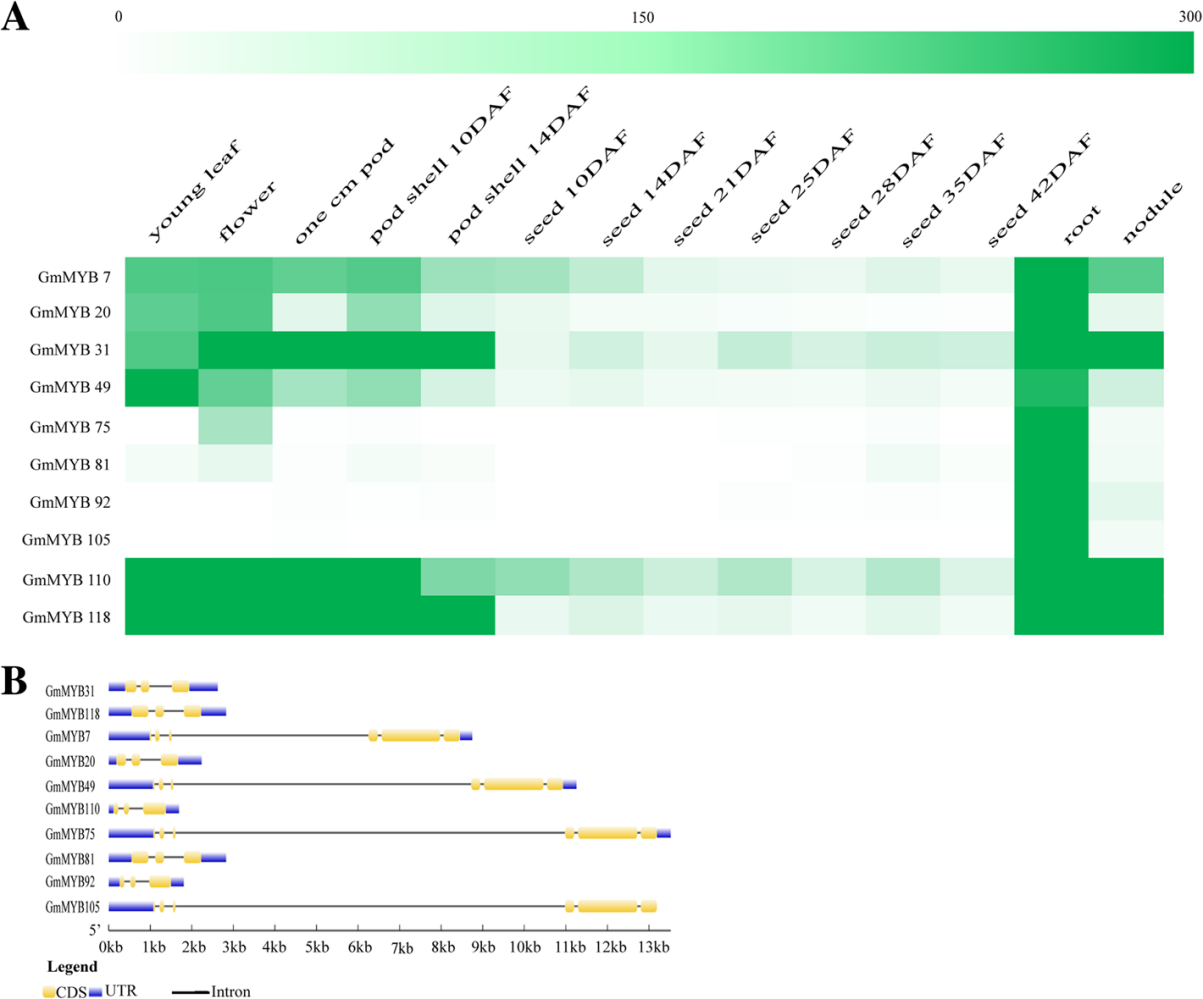
Quantified prediction of tissue expression in soybean and sequence conservation analysis of the ten selected MYB-related TF genes. In accordance with the quantified prediction of fourteen tissue expression provided by SoyBase, the ten MYB-related TF genes that according to the quantified expression level of more than 300 were screened from the 139 MYB-related genes for further analysis. The deeper color represents a greater quantity (**a**). We analyzed the structure using Gene Structure Display Server (http://gsds.cbi.pku.edu.cn/) by submitting CDSs and genomic sequences (**b**)

According to the gene accession number we submitted to the soybase website (http://soybase.org/soyseq/) [10], we obtained the tissue expression data of quantified prediction for a diverse set of fourteen tissue types (Additional file 1: Figure S1). It showed that the expression level of several genes in roots, leaf nodes, leaves and flowers was higher than that in seeds and pods. It may suggest that these genes play a crucial role in soybean growth and development. For further analysis, we screened 10 members from the 139 MYB genes that according to the amount of expression level more than 300 from soybase website prediction in root, including *GmMYB7/20/31/49/75/81/92/105/110/118* (Fig. 3a). It may suggest that these genes play an important role in soybean roots.

### Gene structure analysis of the ten selected MYB-related TFs

To characterize the ten select MYB-related genes, we analyzed their structure using Gene Structure Display Server (http://gsds.cbi.pku.edu.cn/) by submitting coding DNA sequences (CDS) and genomic sequences, and we retrieved basic information (Table 2). As shown in Fig. 3b, the ten MYB-related genes presented with an exon-intron structure. The results showed that the MYB-related genes tended to have closer genetic relationships with more similar structures. For example, *GmMYB7/31/118*, *GmMYB75/92/105* and *GmMYB20/31/110* exhibit similar gene structures, which suggests that they evolved from the same pattern.

**Table 2:** Basic information concerning ten MYB-related genes in soybean

Gene	Gene ID number	Amino acids	p*I*	Molecular mass (kD)	Chromosome	Domain location
*GmMYB7*	Glyma02g03020	300	10.41	32.16	2	94–138
*GmMYB20*	Glyma03g42260	748	6.15	82.07	3	24–68
*GmMYB31*	Glyma05g01640	285	9.66	31	5	80–124
*GmMYB49*	Glyma07g05410	750	6.55	82.3	7	24–68
*GmMYB75*	Glyma11g15180	204	4.48	22.8	11	8–62, 68–113
*GmMYB81*	Glyma12g07110	750	6.2	82.4	12	8–62, 68–113
*GmMYB92*	Glyma13g40830	350	9.09	38.12	13	8–55, 61–106
*GmMYB105*	Glyma15g04620	192	4.61	21.5	15	8–55, 61–106
*GmMYB110*	Glyma16g01980	194	5.07	22.13	16	24–68
*GmMYB118*	Glyma17g10250	194	4.79	22.14	17	144–188

### Promoter regions of the ten MYB-related genes contain various stress-responsive elements

The 2000 bp region upstream of the ATG start codon in the promoters of the ten MYB-related genes was selected. To investigate the mechanism involved in the response to abiotic stresses, plant *cis*-acting elements and PLACE (http://bioinformatics.psb.ugent.be/webtools/plantcare/html/) were used to analyze the regions of the ten gene promoters. A number of regulatory elements that respond to drought and salt stress were identified, including ABRE (ABA-induced), DRE (drought-induced), GT-1 (salt-induced), MYB (drought) and MYC (drought and cold) elements. In addition, the numbers of *cis*-elements for MYB, MYC and GT-1 TFs were greater than other *cis*-elements in these promoters of the ten genes (Table 3). This information revealed that the ten MYB-related genes may be involved in abiotic stress responses, such as drought, salt and cold responses.

**Table 3:** Distribution of *cis*-acting elements within ten MYB-related gene promoters in soybean

Gene	ABRE	DRE	ERE	GARE	GT-1	LTRE	MYB	MYC
*GmMYB7*	9	1	0	1	43	0	14	10
*GmMYB20*	7	0	0	0	23	1	16	20
*GmMYB31*	1	0	0	0	25	0	15	20
*GmMYB49*	16	0	1	0	34	1	20	14
*GmMYB75*	1	4	1	4	28	7	21	24
*GmMYB81*	0	3	0	3	27	5	10	14
*GmMYB92*	7	0	3	1	34	1	20	24
*GmMYB105*	6	0	5	2	27	1	20	22
*GmMYB110*	12	2	1	1	52	3	24	24
*GmMYB118*	3	4	0	3	34	3	16	22

### Several candidates are involved in multiple abiotic stresses

**Fig. 4:**
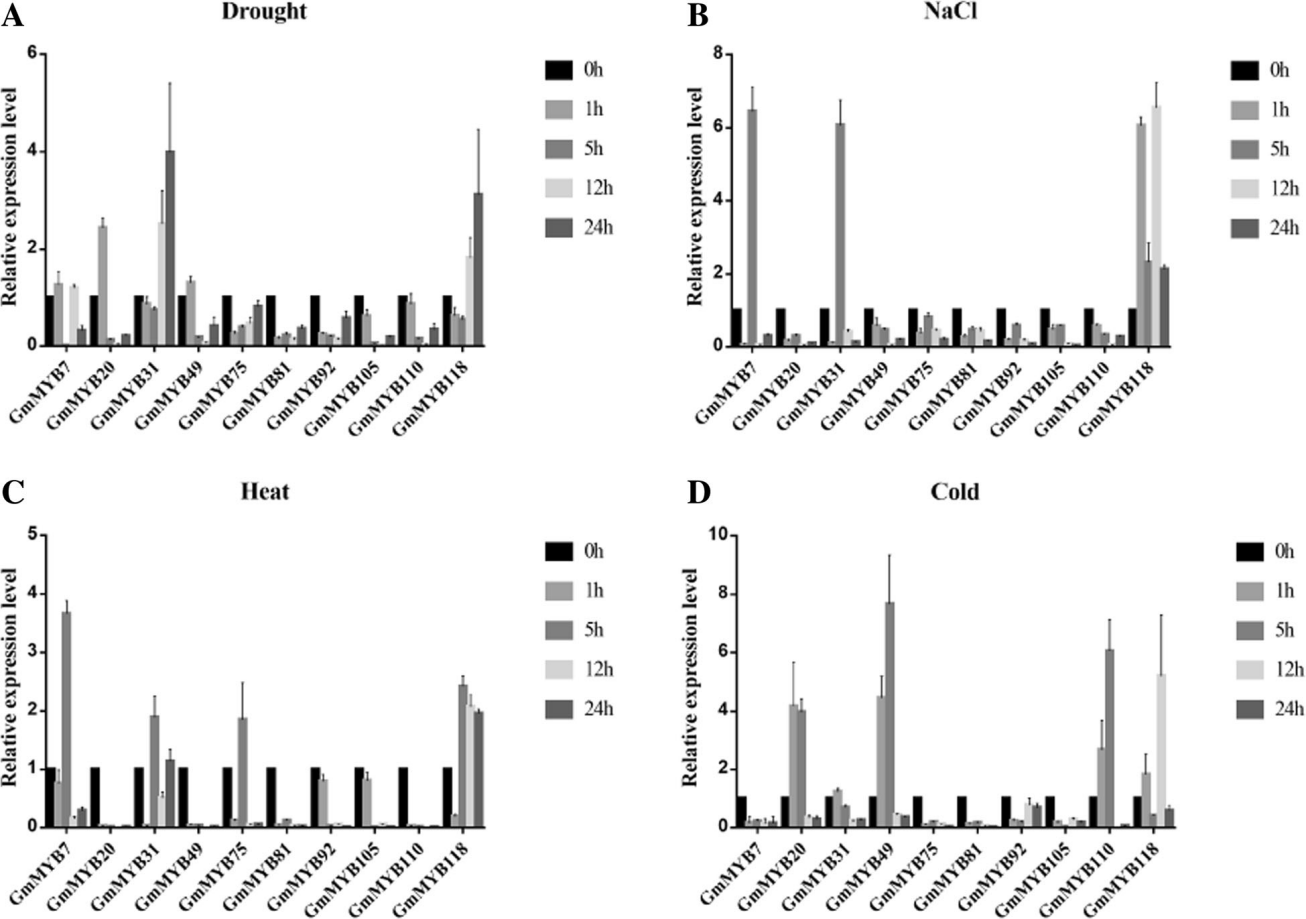
Expression patterns of the ten selected MYB-related TF genes under salt, drought, cold and heat treatment. Fifteen-year-old seedling of soybean were treated with Drought (**a**), NaCl (**b**), Heat (**c**) and Cold (**d**) for 0, 1, 5, 12 and 24 h. The expression patterns of the ten select MYB-related TF genes under various abiotic stresses were quantified by qRT-PCR analysis. *GmMYB118* clearly responded to multiple abiotic stresses including drought, salt, cold and heat stresses (**a**-**d**). The data were shown as the means ± SDs of three experiments

To gain insight into potential functions, we initially examined the expression patterns of the ten MYB-related genes in response to various abiotic stresses by quantitative real-time PCR (qRT-PCR) (Fig. 4). Under drought treatment, the expression of *GmMYB20/31/118* increased by 2.42, 3.98 and 3.11-fold at 1, 5 and 5 h, respectively, the transcription levels of other genes did not change significantly (A). For salt treatment, the expression peaks of *GmMYB7/31/118* occurred at 5, 5 and 12 h, respectively, which were equivalent to 6.45, 6.06 and 6.54-fold increases, respectively. The expression of other genes did not change significantly (B). Under heat treatment, the expression of *GmMYB7/31/75/118* increased by 3.41, 1.96, 1.89 and 2.40-fold at 5 h, respectively, the expression levels of other genes did not change significantly (C). Under cold treatment, the accumulation of *GmMYB20/49/110/118* transcripts increased gradually and peaked at 1, 5, 5 and 12 h; however, the accumulation of *GmMYB118* transcript level was rapidly decreased, it was similar to the CK plants at 12 h. The highest levels of *GmMYB20/49/110/118* were equivalent to 4.16, 7.6, 6.05 and 5.2-fold increases, respectively (D). These results indicated that the accumulation of transcript levels of *GmMYB7/20/31/118* was affected by various abiotic stresses. Among those genes, *GmMYB118* clearly responded to multiple abiotic stresses, including drought, salt, heat and cold (Fig. 4). For this reason, *GmMYB118* was selected for further investigation.

### Subcellular localization of GmMYB118 in *Arabidopsis*

To determine the subcellular localization of GmMYB118, *GmMYB118* was fused to the N-terminus of the humanized green fluorescent protein (hGFP) reporter gene and ligated into an 16318hGFP expression vector under control of the cauliflower mosaic virus (CaMV) 35S promoter. The cDNA coding sequences of *AtWRKY25* (At2g30250) that Located in the nucleus [29] were fused to the N-terminus of the *RFP* gene under the control of the CaMV 35S promoter. Subcellular localization of GFP and RFP expression in *Arabidopsis* mesophyll protoplasts was observed after cotransformation. The *GmMYB118::hGFP* fusion protein localized in the nucleus (Additional file 1: Figure S2A). These observations suggested that GmMYB118 could enter the nucleus to function.

### *GmMYB118* provided drought tolerance in *Arabidopsis*

Overexpression of stress-inducible genes in plants represents an effective strategy for improving abiotic stress tolerance [3, 4, 30–32]. To further investigate the biological functions of the *GmMYB118* gene, three T3 Ectopic expression (EX) lines were selected for analysis under polyethylene glycol (PEG6000) treatment to simulate drought stress. Before conducting the experiment, three-week-old *Arabidopsis* seedlings were subjected to qRT-PCR analysis of *GmMYB118* gene expression in ectopic expression and wild type (WT) plants (Additional file 1: Figure S2B). Expression of *AtActin* was analyzed as a loading control (Additional file 1: Table S1). The relative expression level of *GmMYB118* was equivalent to 8~12 fold in *Arabidopsis*.

For germination assays, seeds of EX and WT lines were germinated on 1/2-strength Murashige and Skoog (MS) media containing various concentrations of PEG6000, and the germination rates was determined at 0, 12, 24, 36, 48, 60 and 72 h. All lines exhibited similar germination rates on 1/2-strength MS media. However, in the presence of PEG6000, the germination of the EX seeds was inhibited, and the degree of inhibition was greater than that of the WT seeds (Additional file 1: Figure S3A). Under normal condition, the germination rate of the WT and EX seeds was about 94~96% at the time points of 72 h (Additional file 1: Figure S3B). Under 3% PEG6000 treatment, the germination rate of the EX seeds was 64.06~72.91%, which was lower than that of the WT seeds (81.77%) at the time points of 24 h (Additional file 1: Figure S3C). Under 6% PEG6000 treatment, the germination rate of the EX seeds was 33.85~34.89%, which was lower than that of the WT seeds 63.02% at the time points of 24 h (Additional file 1: Figure S3D). Under 9% PEG6000 treatment, the germination rate of the EX seeds was 76.56~81.77%, which was lower than that of the WT seeds (94.79%) at the time points of 48 h (Additional file 1: Figure S3E).

**Fig. 5:**
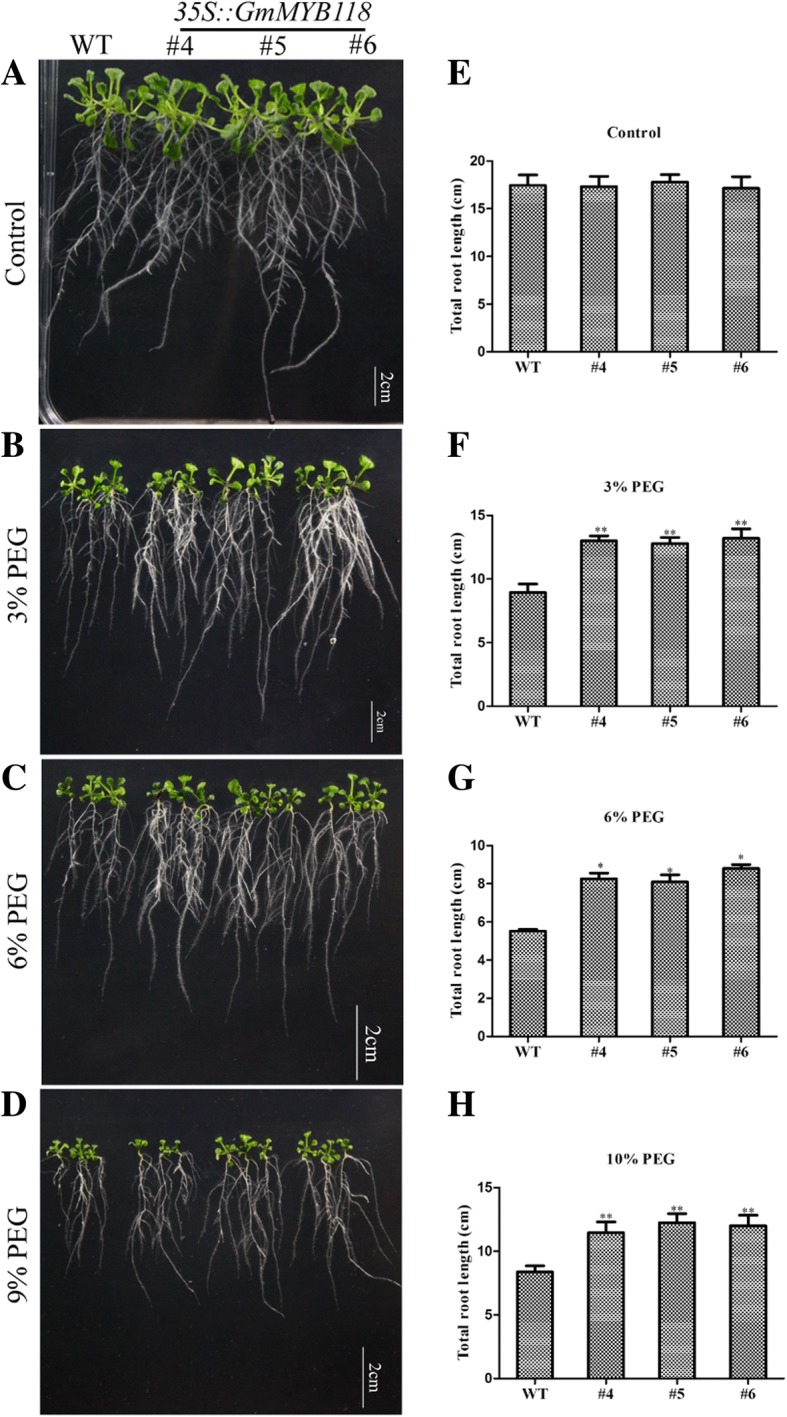
Root length phenotypes of EX lines under PEG treatment. The six-day-old seedlings grown on 1/2 MS were transferred to 1/2 MS medium containing different concentrations of PEG6000. A week later, the growth of roots was photographed of EX and WT (Col-0) seedlings under 0, 3, 6 and 9% PEG6000 treatment (**a**-**d**). Compared with those of the WT (Col-0) seedlings, the statistical results of total root length were shown of the EX seedlings under 0, 3, 6 and 9% PEG treatment (**e**-**h**). The data were shown as the means ± SDs (*n* = 30) of three experiments. ANOVA test demonstrated that there were significant differences (^∗^ *P* < 0.05, ^∗∗^ *P* < 0.01)

For phenotyping of seedlings, the six-day-old *Arabidopsis* seedlings were transferred to 1/2-strength MS medium contained different concentrations of PEG6000 for 7 days. The phenotypes of the transgenic seedlings were similar to those of the WT seedlings under normal conditions (Fig. 5a). As shown in Fig. 5, PEG6000 treatment reduced the root growth of both EX and WT seedlings to some extent (Fig. 5b–d). Under 3 and 9% PEG6000 treatments, the root lengths of the *GmMYB118* lines were 11.86~13.65 cm and 9.34~10.39 cm, respectively, which were significantly longer than those of WT lines (8.38 cm and 6.36 cm, respectively) (Fig. 5f, h). The root length of WT seedlings was also shorter than that EX seedlings under 6% PEG6000 treatment (Fig. 5g). In addition, at the later seedling stage, three-week-old EX and WT seedlings were not watered for 14 days, after which they were pictured after being rewatering 3 days (Fig. 7a). The survival rate of the EX lines after being rewatering 3 days was 90.05~95.63%, which was significantly higher than that of the WT lines (40.50%) (Fig. 7c). These results suggest that *GmMYB118* may potentially function to increase the tolerance of the transgenic plants to drought stress.

### *GmMYB118* provided salt tolerance in *Arabidopsis*

To elucidate the role of the *GmMYB118* in plant growth and development under high salt conditions, salt tolerance experiments involving transgenic and WT lines were carried out. For germination assays, seeds of EX and WT lines were germinated on 1/2-strength MS media that contained various concentrations of NaCl, and the germination rates was determined at 0, 12, 24, 36, 48, 60 and 72 h. Both EX and WT seeds exhibited similar germination rates on 1/2-strength MS media without NaCl (Additional file 1: Figure S4B). In the presence of NaCl, the germination of both the EX and WT seeds was inhibited (Additional file 1: Figure S4A). Under 75 mM NaCl treatment, the germination rate of the EX seeds was 30.18~35.37%, which was lower than that of the WT seeds (55.23%) at the time points of 48 h (Additional file 1: Figure S4C). Under 100 mM NaCl treatment, the germination rate of the EX seeds was 50.47~53.29%, which was lower than that of the WT seeds (69.52%) at the time points of 48 h (Additional file 1: Figure S4D). Under 125 mM NaCl treatment, the inhibition of germination was more severe for the EX seeds than for the WT seeds. The germination rate of the EX seeds ranged from 14.28~18.86%, which was lower than that of the WT seeds (47.62%) (Additional file 1: Figure S4E).

**Fig. 6:**
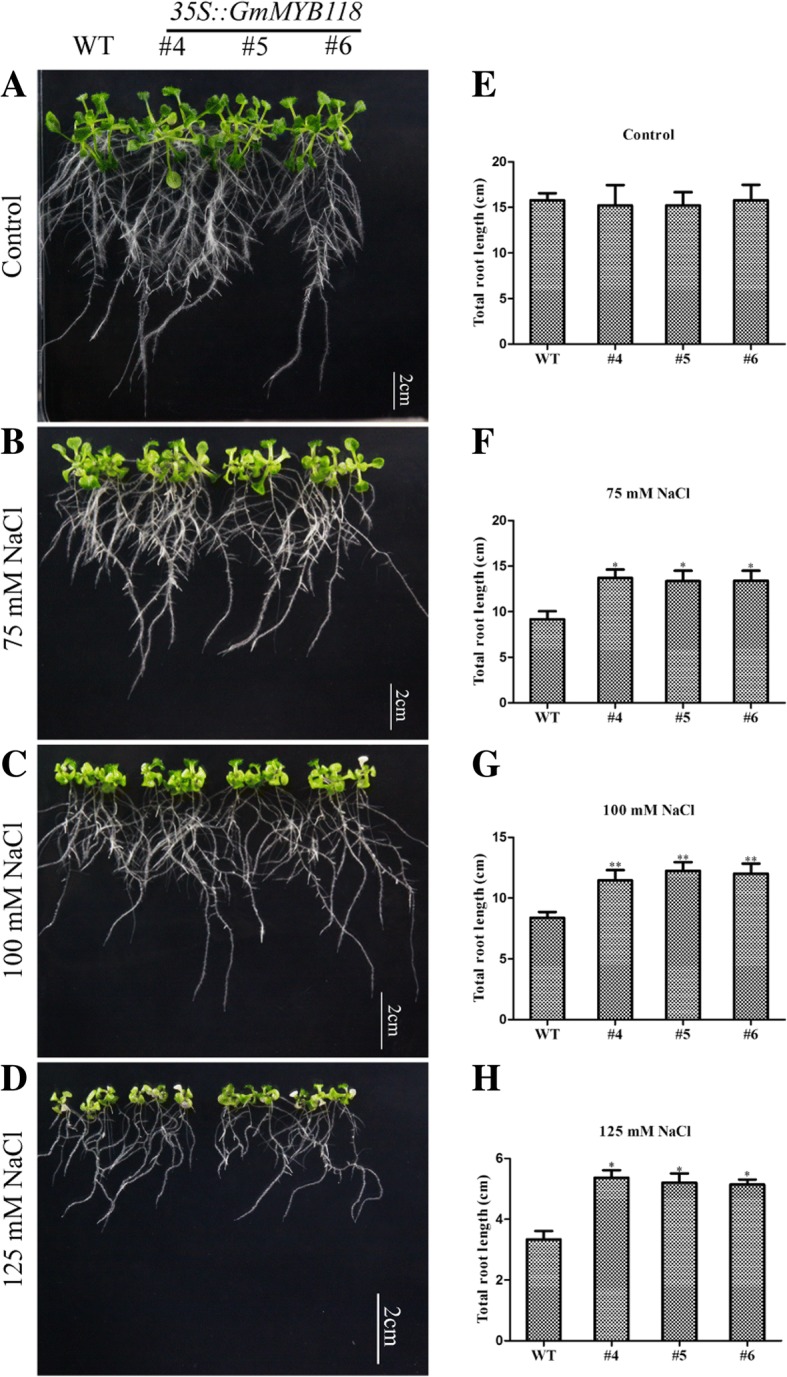
Root length phenotypes of EX lines under NaCl treatment. The six-day-old seedlings grown on 1/2 MS were transferred to 1/2 MS medium containing different concentrations of NaCl. A week later, the growth of roots was photographed of EX and WT (Col-0) seedlings under 0, 75, 100 and 125 mM NaCl treatment (**a**-**d**). Compared with those of the WT seedlings, the statistical results of total root length are shown of the EX seedlings under 0, 75, 100 and 125 mM NaCl treatment (**e**-**h**). The data were shown as the means ± SDs (n = 30) of three experiments. ANOVA test demonstrated that there were significant differences (^∗^ *P* < 0.05, ^∗∗^ *P* < 0.01)

**Fig. 7:**
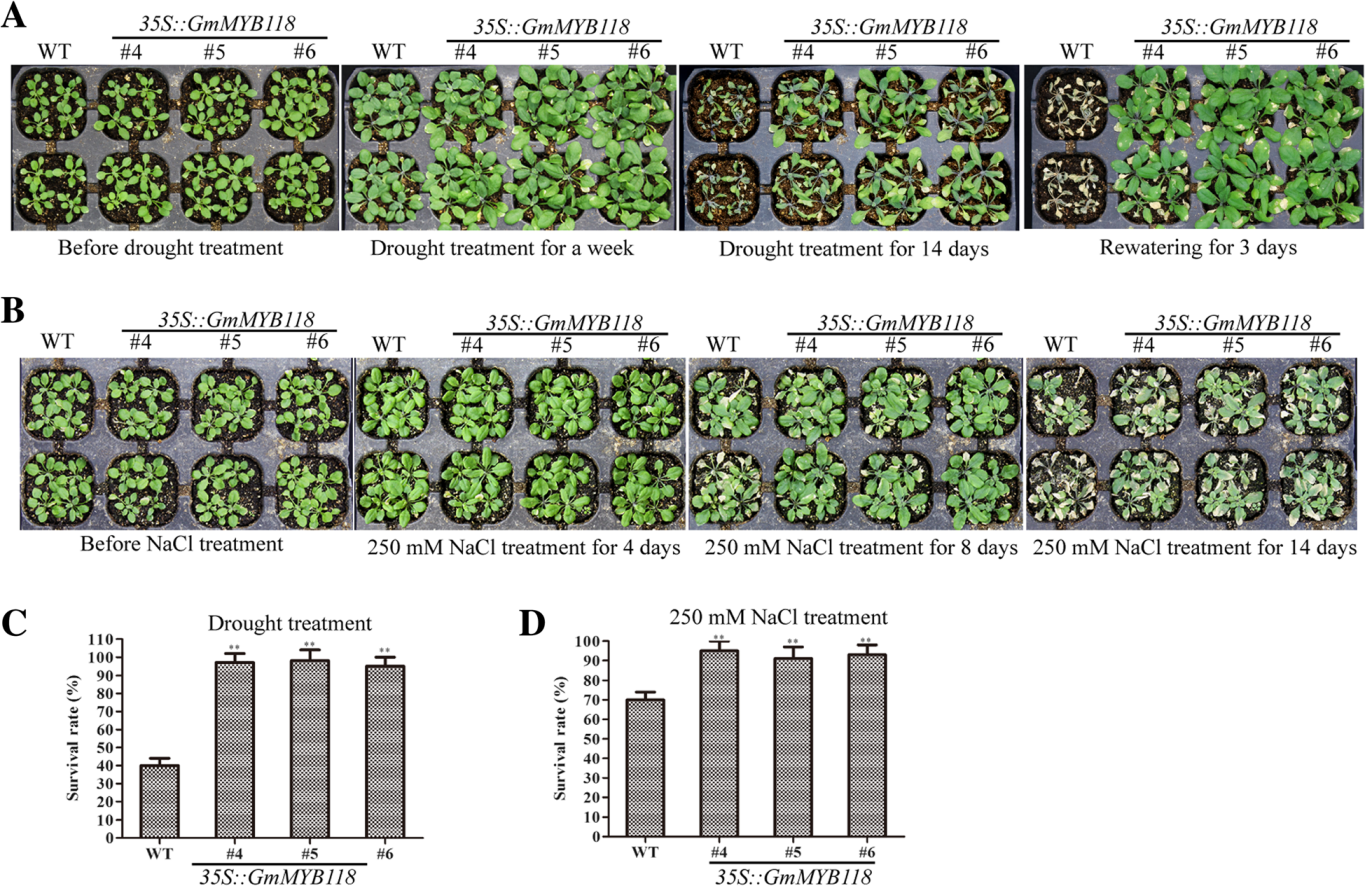
Phenotypes of late-stage EX lines under drought and salt treatments. Three-week-old seedlings were subjected to drought and salt treatment for two weeks. Drought tolerance phenotypes of EX and WT (Col-0) lines under water deficit conditions (**a**). Salinity tolerance phenotypes of EX and WT (Col-0) lines under 250 mM NaCl conditions (**b**). The survival rate of the water-stressed plants was monitored 3 days after rewatering (**c**). The survival rate of the transgenic and WT (Col-0) lines under 250 mM NaCl for 14 days (**d**). The data were shown as the means ± SDs (n = 30) of three experiments. ANOVA test demonstrated that there were significant differences (^∗^ P < 0.05, ^∗∗^ P < 0.01)

For phenotyping, transgenic and WT *Arabidopsis* seeds were grown on 1/2 MS media for 6 days at 22 °C, after which they were transferred to 1/2-strength MS media that contained various concentrations of NaCl and grown for 7 days. The phenotypes of the EX seedlings were similar to those of the WT seedlings under normal conditions (Fig. 6a). As is shown in Fig. 6, under 75, 100 and 125 mM NaCl treatments, the root length of the EX lines was significantly longer than that of the WT lines (Fig. 6b–d). Under 75 and 125 mM NaCl treatments, the total root length of the WT lines (9.39 and 7.69 cm), which was significantly shorter than that of the transgenic lines ranged from 13.11~15.51 and 9.86~10.80 cm (Fig. 6f, h). This difference is most definitive in response to the 100 mM NaCl treatment: the total root length of the EX lines ranged from 11.24~13.51 cm, which was significantly greater than that of the WT lines (8.37 cm) (Fig. 6g). Moreover, at the later seedling stage, three-week-old EX and WT seedlings were grown under 250 mM NaCl for 14 days; their phenotypes are shown in Fig. 7b. The survival rate of the EX lines ranged from 88.32~92.16%, which was significantly greater than that of the WT liens (68.94%) (Fig. 7d). Overall, these results suggested that *GmMYB118* may be used to improve tolerance to salt stress in transgenic plants.

### *GmMYB118* activated stress-responsive genes in *Arabidopsis*

To elucidate the possible molecular mechanisms of the involvement of *GmMYB118* in stress responses, the expression of drought- and salt-responsive marker genes including *AtP5CS1* [33], *AtDREB2A* [34], *AtCOR47* [30], *AtCOR15A* [4], *AtRD29A* [35], *AtKIN1* [36], *AtKIN2* [37], *AtRD22* [38], *AtRAB18* [39], *AtADH1* [40], and *AtNCED3* [41] was investigated in EX lines. A 2-fold change in expression was arbitrarily considered to represent positive expression induction.

**Fig. 8:**
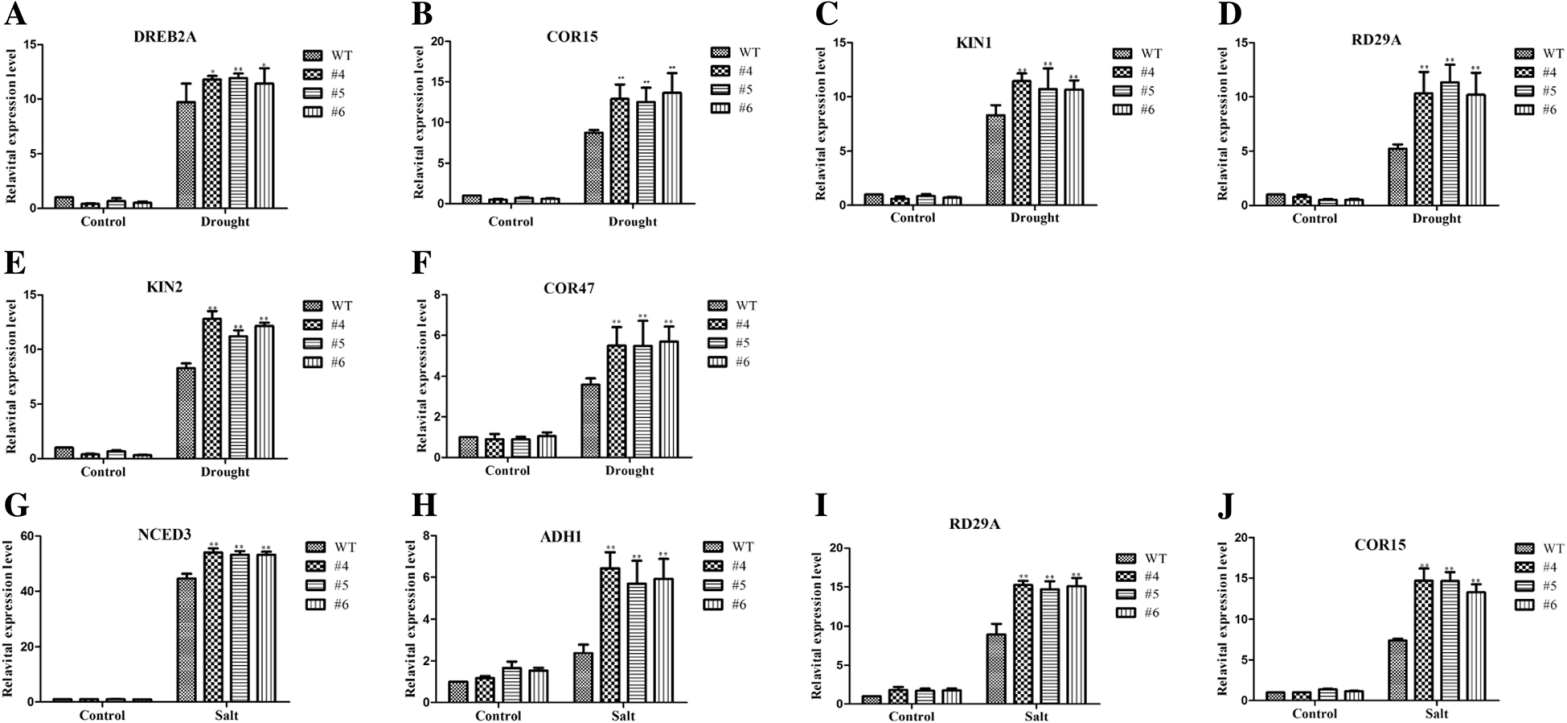
*GmMYB118* regulates stress-responsive gene expression in transgenic *Arabidopsis* plants. Extraction of RNA from two-week-old seedlings grown on 1/2 MS medium with drought and NaCl (100 mM) treatment for 2 h. Gene expression level was quantified by qRT-PCR assays. Expression of *AtActin* was analyzed as a control. Gene-specific primers were used to detect the expression levels of stress-related genes. The expression levels of drought-related genes significantly increased in transgenic *Arabidopsis* plants under drought treatment (**a**-**f**). The expression levels of salt-related genes significantly increased in the transgenic *Arabidopsis* plants under salt treatment (**g**-**j**). The data were the means ± SDs of three experiments. ANOVA test demonstrated that there were significant differences (^∗^ P < 0.05, ^∗∗^ P < 0.01)

qRT-PCR analysis revealed no significant differences at the levels of expression of *AtCOR47*, *AtDREB2A*, *AtKIN1*, *AtKIN2*, *AtRD29A* and *AtCOR15* between the EX lines and WT lines under normal conditions (Fig. 8a–f). Under drought conditions, the expression of these genes in the EX lines significantly higher than that in the WT lines (Fig. 8a–f), although the expression levels of *AtP5CS1* and *AtRAB18* did not differ (data not shown). On the other hand, compared with that in the WT lines, the expression levels of *AtADH1*, *AtNCED3*, *AtCOR15* and *AtRD29A* in the EX lines significantly increased under salt conditions (Fig. 8g–j), but these levels did not markedly differ under normal conditions (Fig. 8g–j). The expression level of *AtRD22* did not significantly differ between the EX lines and the WT lines in either normal or drought conditions (data not shown). These results indicated that overexpression of *GmMYB118* may activate the expression of drought- or salt-responsive genes in *Arabidopsis*, improving the drought and salt stress tolerance of transgenic lines.

### Targeted mutagenesis in soybean hairy roots and GUS staining

To further confirm the functions of the *GmMYB118* gene in soybean, two constructs (pCAMBIA3301-*GmMYB118* and pCas9-GmU6-sgRNA) were generated for overexpression and for gene editing analysis with the CRISPR-Cas9 system (OE and CRISPR constructs, respectively) into soybean hairy roots.

Because the vector of pCAMBIA3301 carries the β-glucuronidase (GUS) reporter gene, we examined the expression level of GUS in accordance with the protocol of a GUS histochemical assay kit to detect the transformation efficiency of the vector by *Agrobacterium rhizogenes (A. rhizogenes)*-mediated transformation. The transformation efficiency was approximately 50% (Additional file 1: Figure S5A). It can be inferred from the results of GUS staining that about 50% of the roots of each OE and CRISPR plant were positive roots. To detect the targeted gene mutations in soybean hairy roots, genomic DNA was collected and extracted for further detection of the target gene mutations in the hairy roots. The target gene was amplified with specific primers and sequenced, and the results showed that some bases were replaced without any insertions or deletions (Additional file 1: Figure S5B). Our results shown that 10% of roots of the coding sequence of *GmMYB118* was edited in each CRISPR plant. The amino acid (I_17_, L_18_, F_19_) of GmMYB118 in 77.5% CRISPR plants was changed, such as from I_17_ to M_17_, L_18_ to A_18_, F_19_ to S_19_. These findings indicated that the CRISPR-Cas9 system modified the gene during hairy root development.

### *GmMYB118* improved drought and salt tolerance in transgenic soybean hairy roots

**Fig. 9:**
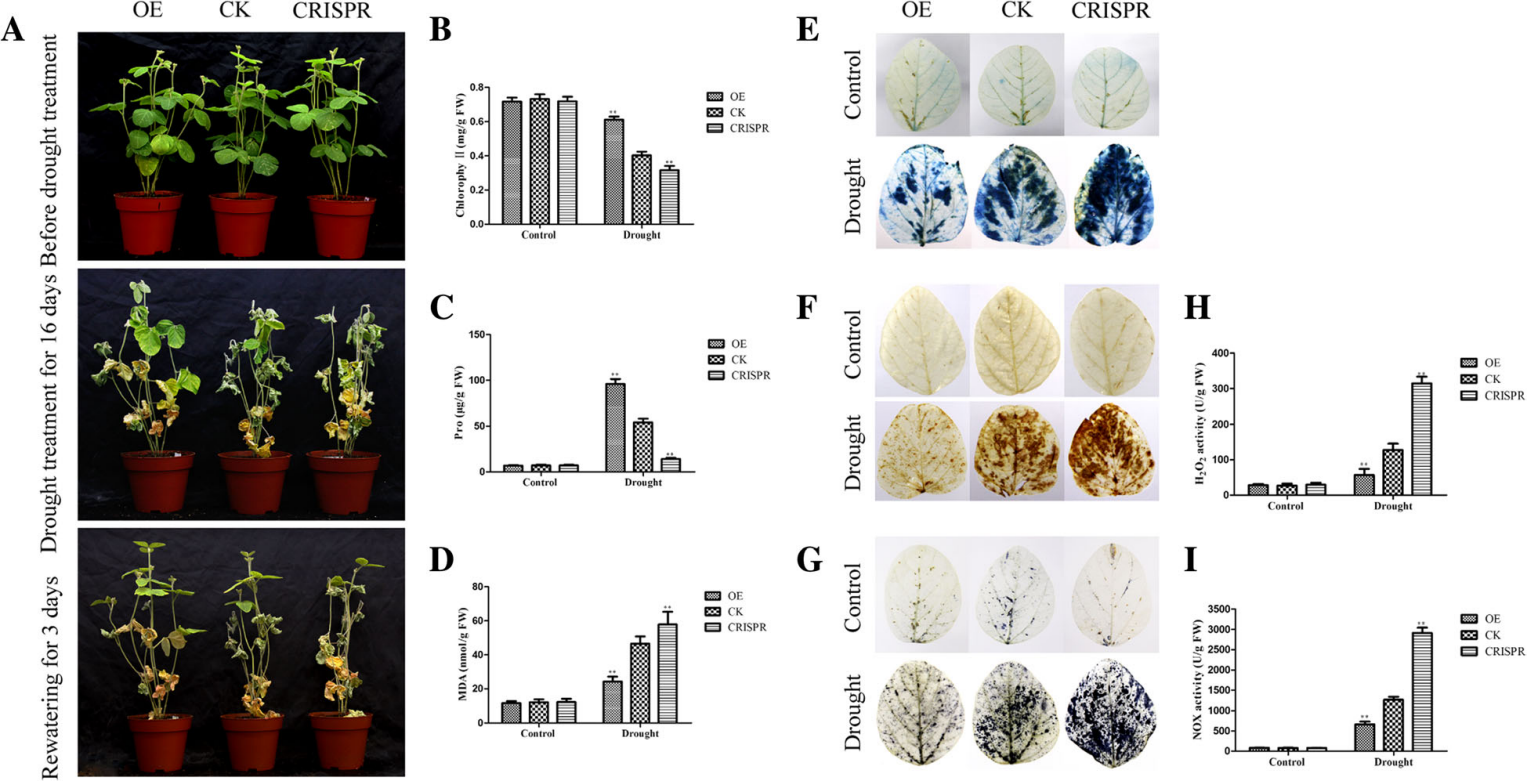
Gm*MYB118* improves drought stress tolerance in transgenic soybean hairy roots. The seedlings with 2–5 cm hairy roots were grown for 5 days on pot under normal condition, then, the plants were dehydrated for 16 days. Survival rate of the water-stressed plants was monitored 3 days after rewatering. Image of drought resistance phenotypes of OE, CK and CRISPR plants under drought conditions was shown (**a**). The leaves contents of Chlorophyll (**b**), Proline (**c**) and MDA (**d**) were detected in OE, CK and CRISPR plants under drought or normal growth condition for a week. Trypan blue staining of soybean plant leaves without irrigation for 14 days (**e**), the dead cells can be strained, but living cells cannot. DAB (**f**) and NBT (**g**) staining of the leaves of OE, CK and CRISPR plants after drought treatment or normal condition for 7 days. The depth of color shows the concentration of H_2_O_2_ and O_2_^−^ in the leaves (**f**-**g**). The content of H_2_O_2_ (**h**) and O_2_^−^ (**i**) in the leaves of OE, CK and CRISPR plants after drought treatment or normal condition for 7 days. The data were means ± SDs of three experiments. ANOVA test demonstrated that there were significant differences (^∗^ P < 0.05, ^∗∗^ P < 0.01)

**Fig. 10:**
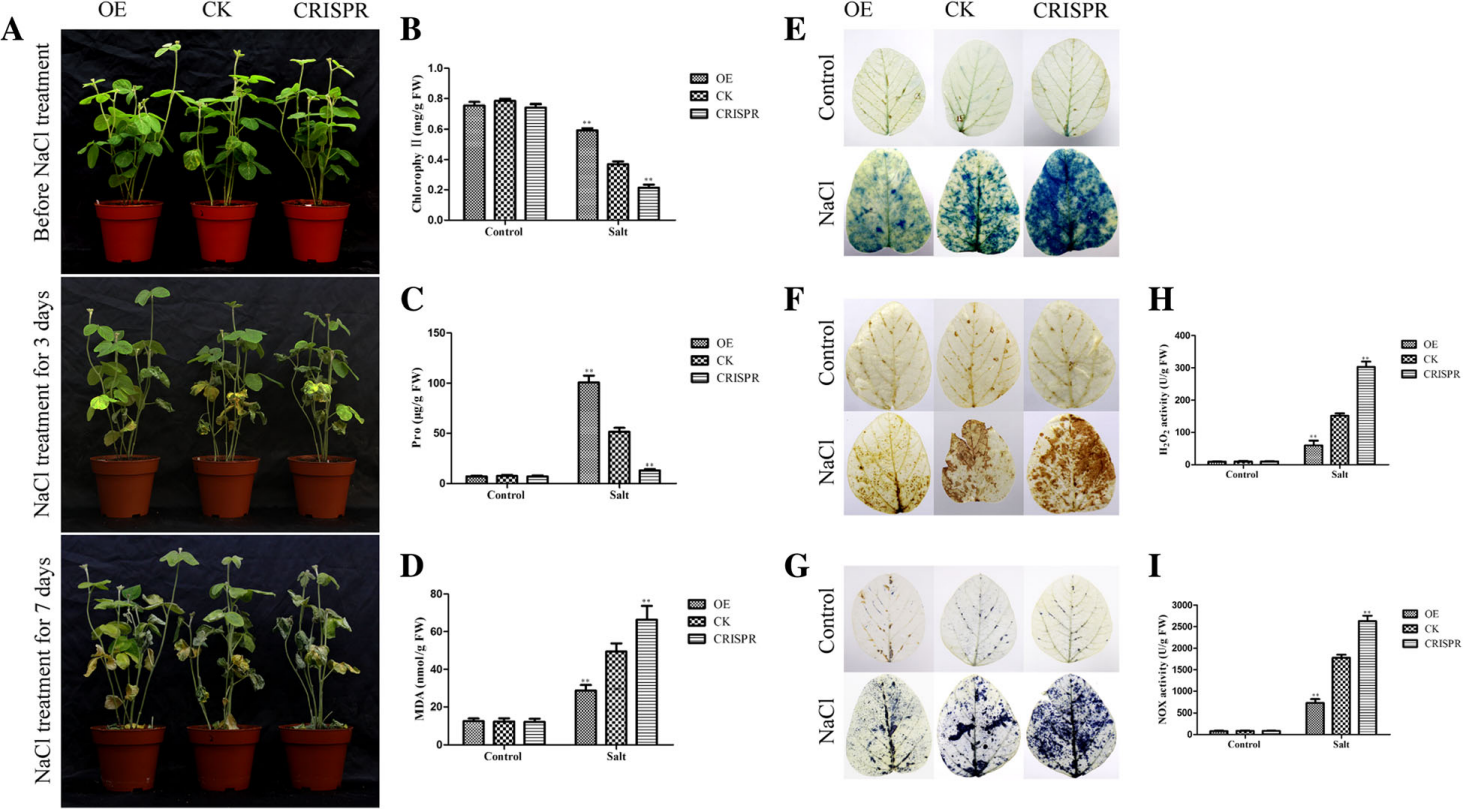
Gm*MYB118* improves salt stress tolerance in transgenic soybean hairy roots. The seedlings with 2–5 cm hairy roots were grown for 5 days on pot under normal condition, then, the plants were 250 mM NaCl treated for a week. Image of salinity resistance phenotypes of OE, CK and CRISPR plants under salt conditions was shown (**a**). The leaves contents of Chlorophyll (**b**), Proline (**c**) and MDA (**d**) were detected in OE, CK and CRISPR plants with 250 mM NaCl treatment or normal growth condition for 3 days. Trypan blue staining of soybean plant leaves without irrigation for a week (**e**), the dead cells can be strained, but living cells cannot. DAB (**f**) and NBT (**g**) staining of the leaves of OE, CK and CRISPR plants after 250 mM NaCl treatment or normal condition for 3 days. The depth of color shows the concentration of H_2_O_2_ and O_2_^−^ in the leaves (**f**-**g**). The content of H_2_O_2_ (**h**) and O_2_^−^ (**i**) in the leaves of OE, CK and CRISPR plants after 250 mM NaCl treatment or normal condition for 3 days. The data were means ± SDs of three experiments. ANOVA test demonstrated that there were significant differences (^∗^ P < 0.05, ^∗∗^ P < 0.01)

The OE and CRISPR lines were analyzed for drought tolerance [1, 25, 42, 43]. For drought treatment, the hairy roots of the seedlings were not watered for 14 days, then rewatering for 3 days. The survival rate of the OE plants was 83.33%, which was clearly greater that of the CK plants (33.33%); however, the survival rate of the CRISPR plants was 16.67%, which was worse than that of the CK plants (Fig. 9a). Similarly, the survival rate of the OE plants was 66.67% under salt conditions, which was clearly greater than that of the CK plants (48.33%). The survival rate of the CRISPR plants was 25.00% lower than that of the CK plants (Fig. 10a).

To investigate the potential physiological mechanism involved in improving the drought resistance of the OE lines, the proline, malondialdehyde (MDA) and chlorophyll contents in the OE, CK and CRISPR plants were measured under both normal growth and stress conditions. The stress condition was described in the method. The proline and chlorophyll contents were 86.41 μg/g and 0.65 mg/g, respectively, which were significantly greater in the OE plants than in the CK plants (47.16 μg/g and 0.39 mg/g, respectively). The proline and chlorophyll contents in the CRISPR plants (16.44 μg/g and 0.29 mg/g, respectively) were evidently lower than those in the CK plants under drought conditions (Fig. 9b, c). Similarly, the proline and chlorophyll contents were 88.17 μg/g and 0.62 mg/g in the OE plants, respectively, and were significantly greater than those in the CK plants (46.70 μg/g and 0.37 mg/g, respectively). The same contents were evidently 12.45 μg/g and 0.20 mg/g lower in the CRISPR plants than in the CK plants under salt conditions, respectively (Fig. 10b, c). Under both drought and salt conditions, the MDA content in the OE plants was lower than that in both the CK and CRISPR plants (Figs. 9d and 10d). By contrast, the MDA contents among all plants did not differ under normal conditions (Figs. 9b–d and 10b–d).

We detected the expression of *GmMYB118* in the hairy roots of transgenic plants subjected to drought and NaCl treatments. Compared with that in the CK plants, the expression in the OE plants increased by 7.9 times, while that of the CRISPR plants decreased by 2.3 times under NaCl treatment. The expression in the OE plants was 5 times greater than that in the CK plants, while the expression in the CRISPR plants was 2 times lower than that in the CK plants under drought treatment (Additional file 1: Figure S6).

### Overexpression of Gm*MYB118* reduced the concentration of O_2_^−^ and H_2_O_2_

Because stress and the intracellular reactive oxygen species (ROS) content affect plant growth and development, we stained soybean leaves with 3,3-diaminobenzidine (DAB) and nitroblue tetrazolium (NBT) to detect H_2_O_2_ and O_2_^−^ contents under normal or stress conditions in OE, CK and CRISPR plants. The stress condition was described in the method. Under normal growth conditions, the DAB and NBT staining of all plant leaves showed no differences (Figs. 9f–g and 10f–g). Under water deficit or the presence of 250 mM NaCl, the color depth of the OE plants was significantly lower than that of the CK plants. In contrast, the color depth of the CRISPR plants was significantly greater than that of the CK plants (Figs. 9f–g and 10f–g). These results suggested that the concentration of H_2_O_2_ and O_2_^−^ in the CK plants was greater than that in the OE plants but lower than that in the CRISPR plants.

The activity of NADPH oxidase (NOX) was closely related to the formation of O_2_^-^, the intermediate product of H_2_O_2_ degradation [18, 44, 45]. Therefore, we measured the concentration of H_2_O_2_ and NOX activity in soybean roots and leaves in accordance with the protocols of an H_2_O_2_ colorimetric assay kit and a NOX assay kit. The results were consistent with the staining results of DAB and NBT; the concentration of H_2_O_2_ and the NOX activity in the CK plants were 54.09 U/g and 600.95 U/g, respectively, which were greater than those in the OE plants (130.77 U/g and 1325.62 U/g, respectively), and the same concentration and activity in the CK plants were lower than those in the CRISPR plants (295.52 U/g and 2896.18 U/g, respectively) under drought conditions (Fig. 9h, i). The concentration of H_2_O_2_ and NOX activity in the CK plants were 52.93 U/g and 641.35 U/g, respectively, which were higher than those in the OE plants (151.15 U/g and 1658.93 U/g, respectively); in addition, the same concentration and activity in the CK plants were lower than those in the CRISPR plants (276.55 U/g and 2530.05 U/g, respectively) under salt conditions (Fig. 10h, i).

In addition, we stained soybean plant leaves with Trypan blue to detect cell activity under normal and stress conditions. As shown in Figs. 9e and 10e, the blue area of the OE plant leaves was obviously smaller than that of the CK plant leaves, and the CK plants were clearly smaller than the CRISPR plants under drought and salt stress conditions. No plant leaves differed under normal growth conditions (Figs. 9e and 10e). These findings suggest that the cell activity in the leaves of the CK plants is lower than that in the leaves of the OE plants but greater than that in the leaves of the CRISPR plants.

## Discussion

In this study, we isolated and identified the *GmMYB118* gene from 139 MYB-related transcription factors. We obtained transgenic *Arabidopsis* and soybean to investigate the potential function of *GmMYB118*. Our results indicated that *GmMYB118* could improve tolerance to drought and salt stresses in *Arabidopsis* and soybean compared to the control lines. In present result, the encoding sequence of *GmMYB118* was edited in the CRISPR hairy roots. Interestingly, the expression level of *GmMYB118* in CRISPR plants was significantly lower than that in CK plants (Additional file 1: Figure S6). It suggests that the stability of mRNA may be affected after editing of *GmMYB118* gene, or the decrease at the expression of *GmMYB118* may be due to the removal or repair mechanism from the host itself after CRISPR editing.

Root is one of the main vegetative organs of plants, which is responsible for absorbing water and minerals dissolved in water, transporting water and minerals to stems and leaves, and storing nutrients [46]. Under the condition of drought and high salt, the root is faced with how to keep water in order to maintain the osmotic balance and to control the ion in and out of the cell membrane to maintain the ion balance, so as to increase the possibility of plant survival. In our result, the expression of *GmMYB118* was the highest in the root (Fig. 3a). No previous studies have shown that the MYB-related gene is the most expressed in the root and performs some certain functions. It can be assumed that *GmMYB118* can improve the osmotic balance of water and the balance of Anions and cations in the cells in the root under stresses conditions, which can directly or indirectly improve the drought resistance and salt tolerance of plants.

Previous studies showed that R2R3-MYB TFs could increase tolerance to various abiotic stresses by participating in many biochemical and physiological processes [14, 47, 48]. Few reports indicated that MYB-related TFs involved in response to abiotic stresses in plants. The MYB-related genes were mainly involved in processes, such as phytochrome regulation, flavonoid biosynthesis, hypocotyl elongation and circadian rhythm [20–23]. Currently, we have found that the expression of *GmMYB118* was induced by drought, salt, heat and cold. Pi et al. reported that *GmMYB173* (*GmMYB118*) interact with the promoter of GmCHS5 in soybean cells to regulate flavonoid biosynthesis [49]. Isoflavones has many biological functions and play an important role in the interaction between plant and environment [47, 50]. Chu et al. was reported that the green and purple leaves of sweet potatoes and the outer leaves of onion possessed higher amounts of flavonoids, and more than 85% of free radical scavenging activities were evaluated [51] It implied that *GmMYB118* was involved in abiotic stresses through regulating of flavonoid biosynthesis. It also suggested that MYB-related TFs could response to abiotic stresses and the processes of flavonoid biosynthesis.

In present study, the experiments of phenotypic and molecular mechanism show that *GmMYB118* improved drought resistance and salt tolerance in soybean with two approaches (OE and CRISPR) through *A. rhizogenes*-mediated transformation system. However, few months ago, Pi et al. reported that the salt-triggered phosphorylation of *GmMYB173*, subsequent increased in its affinity to *GmCHS5* promoter and the elevated expression of *GmCHS5* likely contributed to soybean salt tolerance by enhancing the accumulation of dihydroxy B-ring flavonoids [49]. Unfortunately, under drought condition, we have not found downstream genes directly regulated by *GmMYB118* with some limitations of this study. In the future, it is necessary to investigate whether *GmMYB118* elevate expression of *GmCHS5* to enhance the accumulation of flavonoids in soybean cells under drought condition. It may reveal that whether *GmMYB118* can regulate the same downstream genes to improve tolerance to drought and salt stresses, or different downstream genes to improve drought and salt tolerance. In crop science research, *GmMYB118* can be used as one of the candidate genes for soybean molecular breeding in stresses resistances.

## Conclusion

*GmMYB118* improved tolerance to drought and salt stress by reducing the contents of ROS and MDA.

## Methods

### Identification of MYB-related TFs in soybean

To obtain probable candidate MYB-related TF family members, several sources such as Phytozome (http://www.phytozome.net/) and TFDB (http://planttfdb.cbi.pku.edu.cn/) were accessed to acquire sequence data for bioinformatic analyses of soybean MYB-related TF family members. The resulting protein sequences were then examined for the presence of a MYB motif using the hidden Markov model of the SMART/Pfam tool (http://smart.embl-heidelberg.de/ and http://pfam.xfam.org/). Proteins without a MYB motif were omitted from the datasets. By using alignment and eliminating redundant sequences, we obtained 139 MYB-related TF genes, whose expression was predicted via SoyBase (http://soybase.org/sbt/).

### Chromosomal distribution of MYB-related genes

Chromosomal distribution was investigated using the chromosomal loci in Phytozome. The MapInspect program was used to map chromosomal distributions. The deep blue bars represent the Chrs, and the Chr numbers are shown on the top of the bars. The length of the bar is not represented the size of the Chr. The numbers on the left side of the bars show the distances in megabases (Mb) between neighboring genes.

### Alignment and phylogenetic analysis of MYB-related TFs

Multiple alignment of the amino acid sequences was performed via ClustalX, and the alignments were manually corrected. A phylogenetic tree was constructed with MEGA 6 and the NJ method, and bootstrap analysis with 1000 replicates was used to evaluate the significance of nodes [52].

### Plant materials and stress treatments

Soybean seeds (Tiefeng 8) were germinated for 15 days in pots containing vermiculite. The seedlings were then subjected to various abiotic stresses, including drought, salinity, heat, and cold stresses. For drought stress, the soybean seedlings were placed on filter paper for the induction of rapid drought for 0, 1, 5, 12 and 24 h. For temperature treatments, the soybean seedlings were placed in a 4 °C or 42 °C chamber for cold or heat treatment, respectively, for 0, 1, 5, 12 and 24 h. For salt treatment, the seedlings were transferred to 250 mM NaCl solution for 0, 1, 5, 12 and 24 h. All harvested seedlings were submerged immediately in liquid nitrogen and stored at − 80 °C for RNA extraction.

*Arabidopsis* ecotypes Col-0 was used in this study. Seeds were germinated on 1/2 MS medium with 2% sucrose, after 3 days of vernalization at 4 °C, the plates containing the seeds were housed in a growth chamber that was maintained at a temperature of 22 °C, an irradiance of 40 μmol/m^2^/s^1^, and a photoperiod of 16 h light/8 h dark.

### RNA extraction and qRT-PCR

Trizol reagent was used to extract total RNA in accordance with the manufacturer’s protocol (TIANGEN, China), and the total RNA was treated with DNase I (TaKaRa, Japan) to remove genomic DNA contamination. qRT-PCR was completed with a PrimeScript™ RT Reagent Kit (TaKaRa, Japan) following the manufacturer’s protocol. A pair of gene-specific primers was designed according to soybean MYB-related genes and stress-responsive genes in *Arabidopsis* via Primer Premier 5.0. The *Arabidopsis* and soybean actin gene were used as a control (RT-AtActin and RT-GmActin, Additional file 1: Table S1). qRT-PCR was performed with an ABI Prism 7500 real-time PCR system (ThermoFisher Scientific, USA) equipped with programs in accordance with the methods of Liu [30]. A quantitative analysis was performed using the 2^-ΔΔCT^ method [53]. The primers used for qRT-PCR are listed in Additional file 1: Table S1.

### Vector construction

The coding sequences of *GmMYB118* were amplified by PCR primers (MYB118-F: ATGTCTCGCGCCTCCTC, MYB118-R: AGCAACACTAATGATGCTTTCT). Then, the restriction site (NcoI and BsTEII) in conjunction with gene-specific primers (MYB118–3301, Additional file 1: Table S2) was added to the ends of the *GmMYB118* sequence. The PCR products and pCAMBIA3301 vector were digested with NcoI and BsTEII (ThermoFisher Scientific, USA), after which the products were ligated into pCAMBIA3301 under the control of the CaMV 35S promoter to generate pCAMBIA3301-GmMYB118. For the CRISPR vector, sgRNA seeds of *GmMYB118* were designed by CRISPR-P 2.0 (http://crispr.hzau.edu.cn), which provides web services for computer-aided design of highly efficient sgRNA that exert minimal off-target effects [54]. The sequence of sgRNA seeds was GAACAGTATGATCTCACCGG, it was located in the first exon of the *GmMYB118* gene. The restriction enzyme site (BsaI) sequences (ATTG and AAAC), respectively, was added to the end of the seed and its reverse sequence (sgRNA seeds, Additional file 1: Table S2) to obtain sgRNAs. The pUC57-GmU6 vector was digested completely with BsaI (NEB, USA); afterward, the sgRNAs was ligated into pUC57-GmU6 to obtain pUC57-GmU6-sgRNA. The primer U6-sgRNA (Additional file 1: Table S2) was used to detect whether the sequence is correct or not. The pUC57-GmU6-sgRNA and pCAMBIA3301-Cas9 vectors were digested completely with EcoRI and HindIII (ThermoFisher Scientific, USA) to obtain the fragment of GmU6-sgRNA and the vector was digested, respectively. After digestion, the fragment of GmU6-sgRNA was cloned into the pCAMBIA3301-Cas9 vector with T4 DNA ligase (TransGene, China) to generate pCas9-GmU6-sgRNA vectors. The primer pCas9 (Additional file 1: Table S2) was used to detect whether the sequence is correct or not. All primers are listed in Additional file 1: Table S2.

### *A. rhizogenes*-mediated transformation of soybean hairy roots

To generate transformed soybean hairy roots, the soybean cultivar Williams 82 was used for *A. rhizogenes*-mediated transformation [43]. Seeds were germinated under a 16 h light/8 h dark photoperiod at 25 °C in a humidity chamber. After a week, healthy plants were injected with *A. rhizogenes* strain K599 harboring pCAMBIA3301 (CK) or K599 harboring the construct described above (pCAMBIA3301 or pCas9-GmU6-sgRNA-construct vectors). The infected plants were then transferred to the chamber and kept under high humidity until hairy roots were generated at the infection site and had grown to 2–5 cm in length. The original main roots were removed from the 0.5 cm area below the infection site, then the seedlings with 2–5 cm hairy roots were transferred to pot for 5 days. Afterward, the plants were subjected to drought and 250 mM NaCl treatment for 16 days and 7 days [1, 42].

### Promoter analysis of ten select MYB-related TFs

The 2000 bp region upstream of the ATG start codon of the promoters of MYB family-related genes were selected to identify the *cis*-acting elements by submitting the promoter regions to PLACE (http://bioinformatics.psb.ugent.be/webtools/plantcare/html/). The numbers of each element were then counted manually.

### Trypan blue, DAB and NBT staining

The seedlings with 2–5 cm hairy roots were transferred to pot for 5 days and then subjected to drought (no irrigation) for a week or 250 mM NaCl for 3 days in a growth chamber. Detached leaves from the treated seedlings were stained separately. For DAB staining, the samples were immersed in DAB solution (Solarbio, China) for 12 h and then in 75% ethanol for decoloring until the leaves become white. For NBT staining, the samples were immersed in NBT staining solution (Creek Huizhi, China) for 12 h and then in 75% ethanol [18] decoloring until the leaves become white. For Trypan blue staining, differently, the plants were subjected to drought for 16 days. The samples were immersed in 0.5% Trypan blue (BioDee, China) solution for 12 h and then in 75% ethanol for decoloring until the leaves become white. Images were taken with Canon 50D (Canon, Japan) camera.

### Quantification of the H_2_O_2_ content and NOX activity

Prior to H_2_O_2_ measurements, the soybean plants that transferred to pot for 5 days were subjected to drought and 250 mM NaCl stress for a week and 3 days. Afterward, the H_2_O_2_ content of leaves was determined in accordance with the protocol of an H_2_O_2_ colorimetric assay kit (Beyotime, China) [18]. Similarly, the NOX activity of leaves was determined with a NOX assay kit (Solarbio, China) in accordance with the manufacturer’s protocol. All the measurements were repeated three times, and ANOVA test was used for statistical analysis.

### Subcellular localization assays

The full-length cDNA sequences of *GmMYB118* were fused to the N-terminus of the *hGFP* gene (MYB118-GFP, Additional file 1: Table S2) under the control of the CaMV 35S promoter. The cDNA coding sequences of *AtWRKY25* (At2g30250) that Located in the nucleus [29] were fused to the N-terminus of the *mCherry* gene (WRKY25-RFP, Additional file 1: Table S2) under the control of the CaMV 35S promoter. The recombinant plasmid of *GmMYB118-*GFP and *AtWRKY25-RFP* were cotransformed into *Arabidopsis* protoplasts via the PEG4000-mediated method [18, 55]. The expression of the fusion protein was observed under dark conditions for 12 h, and GFP and RFP was detected by laser scanning confocal microscopy (Zeiss LSM 700, Germany) [18, 30].

### Drought and salt stress assays of transgenic *Arabidopsis* plants

To obtain EX plants, the full-length cDNA sequence of *GmMYB118* was introduced into a pCAMBIA1302 plant transformation vector (MYB118–3301, Additional file 1: Table S2). Recombinant vectors were confirmed by sequencing, after which they were then transformed into *Agrobacterium tumefaciens* (GV3101). WT *Arabidopsis thaliana* (Col-0) plants were then infected with the transformed bacteria by the floral dip method [56].

The seeds of WT and EX (independent transgenic lines 4, 5 and 6) lines were disinfected with sodium hypochlorite. After 3 days of vernalization at 4 °C, the plates containing the seeds were transferred to a growth chamber. Three-week-old *Arabidopsis* seedlings were subjected to qRT-PCR analysis of *GmMYB118* gene expression in ectopic expression and WT (Col-0) plants (Additional file 1: Figure S2B). Expression of *AtActin* was analyzed as a loading control (Additional file 1: Table S1).

For germination assays, approximately 80 sterilized seeds of every genotype of the WT and EX plants were sown on 1/2-strength MS growth media that were supplemented with various concentrations of PEG6000 (0, 3, 6 and 9%) (Merck, USA) or NaCl (0, 75, 100 and 125 mM) (XiLONG, China). The plates were housed in a growth chamber that was maintained at a temperature of 22 °C, an irradiance of 40 μmol/m^2^/s^1^, and a photoperiod of 16 h light/8 h dark, as described previously [57, 58]. The number of germinated seeds was counted every 12 h, and at least 80 seeds per genotype were measured.

For root growth assays, sterilized WT and EX seeds were sown on 1/2-strength MS growth media. Five-day-old seedlings were transferred to growth media that contained different concentrations of PEG6000 (0, 3, 6 and 9%) (Merck, USA) or NaCl (0, 75, 100 and 125 mM) (XiLONG, China) for a week. Images were collected after 7 days of growth, and the root lengths were evaluated via an Epson Expression 11000XL root system scanning analyzer (Epson, Japan) [57]. At least 30 seedlings per genotype were measured.

To test drought and salt tolerance at later developmental stages, three-week-old seedlings were subjected to dehydration or 250 mM NaCl for 14 days. The plant phenotypes were imaged, and the plants were counted to determine the survival rate. At least 30 seedlings were measured per line in each treatment, and all stress assays were performed at least three times.

### Heat and freezing stress assays of transgenic *Arabidopsis* plants

To test the heat tolerance at the seedling stage, sterilized WT and EX seeds were sown on 1/2-strength MS growth media. Five-day-old seedlings were subjected to 37 °C for 1 h, allowed to recover at 22 °C for 2 h, and then subjected to 44 °C for 4.5 h [59]. For freezing tolerance assays, 5-day-old seedlings were subjected to − 4 °C for 4 h [34]. After the seedlings recovered for 5 days, their phenotypes were imaged, and the plants were counted to determine the survival rate. At least 60 seedlings were measured per line in each treatment, and all stress assays were performed at least three times.

### Measurements of proline and MDA contents

Prior to measurements, the soybean plants that transferred into pot for 5 days were subjected to drought or 250 mM NaCl stress for a week or 3 days, after which the proline content of leaves was measured as described previously [60]. Similarly, the MDA content of leaves was determined with an MDA assay kit (Comin, China) in accordance with the manufacturer’s protocol. All the measurements were repeated three times, and ANOVA test was used for statistical analyses.

## Additional file

Additional file 1: For disinfection of Arabidopsis thaliana seeds, the seeds of WT and EX (independent transgenic lines 4, 5 and 6) lines was disinfected with sodium hypochlorite. After 3 days of vernalization at 4 °C, the plates containing the seeds was transferred to a growth chamber. For statistical methods of data, the data shown are the means ± SDs (n = 80) of three experiments. ANOVA tests demonstrated that there were significant differences (∗ *P* < 0.05, ∗∗ ^P^ < 0.01). Expression of *AtActin* was analyzed as a loading control in *Arabidopsis* (**Table S1**). Expression of *GmActin* was analyzed as a control in soybean (**Table S1.**) **Figure S1.** Quantitative gene expression of all MYB-related TF family members in soybean. The tissue expression data of quantified prediction for a diverse set of fourteen tissue types from the soybase website (http://soybase.org/soyseq/).** Figure S2.** Subcellular localization and expression level of GmMYB118. GmMYB118 was localized in the nucleus in Arabidopsis mesophyll protoplasts (A). Scale bars = 20 μm. *GmMYB118* gene expression in ectopic expression and WT (Col-0) plants (B). **Figure S3.** Germination rate of OE lines under PEG treatment. Images of germinating EX and WT (Col-0) seeds after 72 h under 0, 3, 6 and 9% PEG6000 treatment (A). The germination of the WT and EX plants of sown on 1/2-strength MS growth media with 0% (B), 3% (C), 6% (D) and 9% PEG6000 (E) were monitored until to 72 h. **Figure S4.** Germination rate of EX lines under NaCl treatment. Images of germinating EX and WT (Col-0) seeds after 72 h under 0, 75, 100 and 125 mM NaCl treatment (A). The germination of the WT and EX plants of sown on 1/2-strength MS growth media with 0 (B), 75 (C), 100 (D) and 125 mM NaCl (E) were monitored until to 72 h. **Figure S5.** Targeted mutagenesis in soybean hairy roots and GUS staining. The GUS staining of transgenic hairy roots revealed a transformation efficiency of approximately 50% (A). The target gene was amplified with specific primers and sequenced (AuGCT, China). The results show that some bases have been replaced (B). **Figure S6.** Expression level of *GmMYB118* under drought and salt treatments. Expression level of *GmMYB118* under drought (B) and salt (C) treatments was quantified by qRT-PCR assays. The expression level of *GmMYB118* under normal condition was shown in **Figure S6A.** **Table S1.** Gene-specific primers used for qRT-PCR. **Table S2**. Primers used to construct recombinant vectors. (PDF 753 kb)

## Abbreviations

*A. rhizogenes*: *Agrobacterium rhizogenes*
ABA: Abscisic acid
Chr: Chromosome
DAB: 3,3-diaminobenzidine
DBD: DNA-binding domain
GUS: β-glucuronidase
MDA: Malondialdehyde
NBT: Nitroblue tetrazolium
NOX: NADPH oxidase
PEG: Polyethylene glycol
qRT-PCR: Quantitative real-time PCR
ROS: Reactive oxygen species
TF: Transcription factor
WT: Wild type

## References

[1] Wang F, Chen HW, Li QT, Wei W, Li W, Zhang WK, Ma B, Bi YD, Lai YC, Liu XL (2015). GmWRKY27 interacts with GmMYB174 to reduce expression of GmNAC29 for stress tolerance in soybean plants. Plant J.

[2] Yamaguchi-Shinozaki K, Shinozaki K (2006). Transcriptional regulatory networks in cellular responses and tolerance to dehydration and cold stresses. Annu Rev Plant Biol.

[3] Xu ZS, Chen M, Li LC, Ma YZ (2008). Functions of the ERF transcription factor family in plants. Botany.

[4] Xu ZS, Chen M, Li LC, Ma YZ (2011). Functions and application of the AP2/ERF transcription factor family in crop improvement. J Integr Plant Biol.

[5] Yanhui C, Xiaoyuan Y, Kun H, Meihua L, Jigang L, Zhaofeng G, Zhiqiang L, Yunfei Z, Xiaoxiao W, Xiaoming Q (2006). The MYB transcription factor superfamily of Arabidopsis: expression analysis and phylogenetic comparison with the rice MYB family. Plant Mol Biol.

[6] Zhao Y, Tian X, Wang F, Zhang L, Xin M, Hu Z, Yao Y, Ni Z, Sun Q, Peng H (2017). Characterization of wheat MYB genes responsive to high temperatures. BMC Plant Biol.

[7] Chu S, Wang J, Zhu Y, Liu S, Zhou X, Zhang H, Wang CE, Yang W, Tian Z, Cheng H (2017). An R2R3-type MYB transcription factor, GmMYB29, regulates isoflavone biosynthesis in soybean. PLoS Genet.

[8] Liu L (2008). The roles of MYB transcription factors on plant defense responses and its molecular mechanism. Hereditas (Beijing).

[9] Martin C, PazAres J (1997). MYB transcription factors in plants. Trends Genet.

[10] Bian Shaomin, Jin Donghao, Li Ruihua, Xie Xin, Gao Guoli, Sun Weikang, Li Yuejia, Zhai Lulu, Li Xuyan (2017). Genome-Wide Analysis of CCA1-Like Proteins in Soybean and Functional Characterization of GmMYB138a. International Journal of Molecular Sciences.

[11] Wang F, Suo Y, Wei H, Li M, Xie C, Wang L, Chen X, Zhang Z (2015). Identification and characterization of 40 isolated Rehmannia glutinosa MYB family genes and their expression profiles in response to shading and continuous cropping. Int J Mol Sci.

[12] Xiong H, Li J, Liu P, Duan J, Zhao Y, Guo X, Li Y, Zhang H, Ali J, Li Z (2014). Overexpression of OsMYB48-1, a novel MYB-related transcription factor, enhances drought and salinity tolerance in rice. PLoS One.

[13] Baldoni E, Genga A, Cominelli E (2015). Plant MYB transcription factors: their role in drought response mechanisms. Int J Mol Sci.

[14] Dubos C, Stracke R, Grotewold E, Weisshaar B, Martin C, Lepiniec L (2010). MYB transcription factors in Arabidopsis. Trends Plant Sci.

[15] Feng G, Burleigh JG, Braun EL, Mei W, Barbazuk WB (2017). Evolution of the 3R-MYB gene family in plants. Genome Biol Evol.

[16] Zhang L, Zhao G, Xia C, Jia J, Liu X, Kong X (2012). Overexpression of a wheat MYB transcription factor gene, TaMYB56-B, enhances tolerances to freezing and salt stresses in transgenic Arabidopsis. Gene.

[17] Xu R, Wang Y, Zheng H, Lu W, Wu C, Huang J, Yan K, Yang G, Zheng C (2015). Salt-induced transcription factor MYB74 is regulated by the RNA-directed DNA methylation pathway in Arabidopsis. J Exp Bot.

[18] Wang N, Zhang WX, Qin MY, Li S, Qiao M, Liu ZH, Xiang FN (2017). Drought tolerance conferred in soybean (Glycine max. L) by GmMYB84, a novel R2R3-MYB transcription factor. Plant Cell Physiol.

[19] Chen N, Yang Q, Pan L, Chi X, Chen M, Hu D, Yang Z, Wang T, Wang M, Yu S (2014). Identification of 30 MYB transcription factor genes and analysis of their expression during abiotic stress in peanut (Arachis hypogaea L.). Gene.

[20] Kobayashi S, Ishimaru M, Hiraoka K, Honda C (2002). Myb-related genes of the Kyoho grape ( Vitis labruscana) regulate anthocyanin biosynthesis. Planta.

[21] Lee MM, Schiefelbein J (1999). WEREWOLF, a MYB-related protein in Arabidopsis, is a position-dependent regulator of epidermal cell patterning. Cell.

[22] Noda K, Glover BJ, Linstead P, Martin C (1994). Flower colour intensity depends on specialized cell shape controlled by a Myb-related transcription factor. Nature.

[23] Nguyen Nguyen Hoai, Lee Hojoung (2016). MYB-related transcription factors function as regulators of the circadian clock and anthocyanin biosynthesis inArabidopsis. Plant Signaling & Behavior.

[24] Glover BJ, Perez-Rodriguez M, Martin C (1998). Development of several epidermal cell types can be specified by the same MYB-related plant transcription factor. Development.

[25] Yi J, Derynck MR, Li X, Telmer P, Marsolais F, Dhaubhadel S (2010). A single-repeat MYB transcription factor, GmMYB176, regulates CHS8 gene expression and affects isoflavonoid biosynthesis in soybean. Plant J.

[26] Urao T, Noji M, YamaguchiShinozaki K, Shinozaki K (1996). A transcriptional activation domain of ATMYB2, a drought-inducible Arabidopsis Myb-related protein. Plant J.

[27] Schmutz J, Cannon SB, Schlueter J, Ma J, Mitros T, Nelson W, Hyten DL, Song Q, Thelen JJ, Cheng J (2010). Genome sequence of the palaeopolyploid soybean. Nature.

[28] Du H, Wang YB, Xie Y, Liang Z, Jiang SJ, Zhang SS, Huang YB, Tang YX (2013). Genome-wide identification and evolutionary and expression analyses of MYB-related genes in land plants. DNA Res.

[29] Jiang YQ, Deyholos MK (2009). Functional characterization of Arabidopsis NaCl-inducible WRKY25 and WRKY33 transcription factors in abiotic stresses. Plant Mol Biol.

[30] Liu P, Xu ZS, Lu PP, Hu D, Chen M, Li LC, Ma YZ (2013). A wheat PI4K gene whose product possesses threonine autophophorylation activity confers tolerance to drought and salt in Arabidopsis. J Exp Bot.

[31] Riechmann JL, Heard J, Martin G, Reuber L, Jiang CZ, Keddie J, Adam L, Pineda O, Ratcliffe OJ, Samaha RR (2000). Arabidopsis transcription factors: genome-wide comparative analysis among eukaryotes. Science.

[32] Flowers TJ (2004). Improving crop salt tolerance. J Exp Bot.

[33] Cheng L, Li X, Huang X, Ma T, Liang Y, Ma X, Peng X, Jia J, Chen S, Chen Y (2013). Overexpression of sheepgrass R1-MYB transcription factor LcMYB1 confers salt tolerance in transgenic Arabidopsis. Plant Physiol Biochem.

[34] Liu Q, Kasuga M, Sakuma Y, Abe H, Miura S, Yamaguchi-Shinozaki K, Shinozaki K, Baek D (1998). Two transcription factors, DREB1 and DREB2, with an EREBP/AP2 DNA binding domain separate two cellular signal transduction pathways in drought- and low-temperature-responsive gene expression, respectively, in Arabidopsis. Plant Cell.

[35] Yamaguchi-Shinozaki Kazuko, Shinozaki Kazuo (1994). A Novel cis-Acting Element in an Arabidopsis Gene Is Involved in Responsiveness to Drought, Low-Temperature, or High-Salt Stress. The Plant Cell.

[36] Kurkela S, Franck M (1990). Cloning and characterization of a cold- and ABA-inducible Arabidopsis gene. Plant Mol Biol.

[37] Kurkela S, Borg-Franck M (1992). Structure and expression of kin2, one of two cold- and ABA-induced genes of Arabidopsis thaliana. Plant Mol Biol.

[38] Harshavardhan VT, Van Son L, Seiler C, Junker A, Weigelt-Fischer K, Klukas C, Altmann T, Sreenivasulu N, Baumlein H, Kuhlmann M (2014). AtRD22 and AtUSPL1, members of the plant-specific BURP domain family involved in Arabidopsis thaliana drought tolerance. PLoS One.

[39] Lang V, Palva ET (1992). The expression of a Rab-related gene, rab18, is induced by abscisic acid during the cold acclimation process of Arabidopsis thaliana (L.) Heynh. Plant Mol Biol.

[40] Hoeren FU, Dolferus R, Wu Y, Peacock WJ, Dennis ES (1998). Evidence for a role for AtMYB2 in the induction of the Arabidopsis alcohol dehydrogenase gene (ADH1) by low oxygen. Genetics.

[41] Ruggiero B, Koiwa H, Manabe Y, Quist TM, Inan G, Saccardo F, Joly RJ, Hasegawa PM, Bressan RA, Maggio A (2004). Uncoupling the effects of abscisic acid on plant growth and water relations. Analysis of sto1/nced3, an abscisic acid-deficient but salt stress-tolerant mutant in arabidopsis. Plant Physiol.

[42] Sun X, Hu Z, Chen R, Jiang Q, Song G, Zhang H, Xi Y (2015). Targeted mutagenesis in soybean using the CRISPR-Cas9 system. Sci Rep.

[43] Kereszt A, Li DX, Indrasumunar A, Nguyen CDT, Nontachaiyapoom S, Kinkema M, Gresshoff PM (2007). Agrobacterium rhizogenes - mediated transformation of soybean to study root biology. Nat Protoc.

[44] Mittler R, Vanderauwera S, Gollery M, Van Breusegem F (2004). Reactive oxygen gene network of plants. Trends Plant Sci.

[45] Foreman J, Demidchik V, Bothwell JHF, Mylona P, Miedema H, Torres MA, Linstead P, Costa S, Brownlee C, Jones JDG (2003). Reactive oxygen species produced by NADPH oxidase regulate plant cell growth. Nature.

[46] Schachtman DP, Goodger JQD (2008). Chemical root to shoot signaling under drought. Trends Plant Sci.

[47] Czemmel S, Heppel SC, Bogs J (2012). R2R3 MYB transcription factors: key regulators of the flavonoid biosynthetic pathway in grapevine. Protoplasma.

[48] Du H, Yang SS, Liang Z, Feng BR, Liu L, Huang YB, Tang YX (2012). Genome-wide analysis of the MYB transcription factor superfamily in soybean. BMC Plant Biol.

[49] Pi E, Zhu C, Fan W, Huang Y, Qu L, Li Y, Zhao Q, Ding F, Qiu L, Wang H (2018). Quantitative Phosphoproteomic and Metabonomic Analyses Reveal GmMYB173 Optimizes Flavonoid Metabolism in Soybean under Salt Stress. MCP Papers in Press.

[50] Koes RE, Quattrocchio F, Mol JNM (1994). The flavonoid biosynthetic pathway in plants: function and evolution. Bioessays.

[51] Chu YH, Chang CL, Hsiafen H (2000). Flavonoid content of several vegetables and their antioxidant activity. J Sci Food Agric.

[52] Tamura K, Stecher G, Peterson D, Filipski A, Kumar S (2013). MEGA6: molecular evolutionary genetics analysis version 6.0. Mol Biol Evol.

[53] Le DT, Nishiyama R, Watanabe Y, Mochida K, Yamaguchi-Shinozaki K, Shinozaki K, Tran LS (2011). Genome-wide expression profiling of soybean two-component system genes in soybean root and shoot tissues under dehydration stress. DNA Res.

[54] Lei Y, Lu L, Liu HY, Li S, Xing F, Chen LL (2014). CRISPR-P: a web tool for synthetic single-guide RNA design of CRISPR-system in plants. Mol Plant.

[55] He GH, Xu JY, Wang YX, Liu JM, Li PS, Chen M, Ma YZ, Xu ZS (2016). Drought-responsive WRKY transcription factor genes TaWRKY1 and TaWRKY33 from wheat confer drought and/or heat resistance in Arabidopsis. BMC Plant Biol.

[56] Clough SJ, Bent AF (1998). Floral dip: a simplified method for agrobacterium-mediated transformation of Arabidopsis thaliana. Plant J.

[57] Zhao SP, Xu ZS, Zheng WJ, Zhao W, Wang YX, Yu TF, Chen M, Zhou YB, Min DH, Ma YZ (2017). Genome-wide analysis of the RAV family in soybean and functional identification of GmRAV-03 involvement in salt and drought stresses and exogenous ABA treatment. Front Plant Sci.

[58] Feng CZ, Chen Y, Wang C, Kong YH, Wu WH, Chen YF (2014). Arabidopsis RAV1 transcription factor, phosphorylated by SnRK2 kinases, regulates the expression of ABI3, ABI4, and ABI5 during seed germination and early seedling development. Plant J.

[59] Li SX, Liu JX, Liu ZY, Li XR, Wu FJ, He YK (2014). HEAT-INDUCED TAS1 TARGET1 mediates Thermotolerance via HEAT STRESS TRANSCRIPTION FACTOR A1a-directed pathways in Arabidopsis. Plant Cell.

[60] Bu QY, Lv TX, Shen H, Luong P, Wang J, Wang ZY, Huang ZG, Xiao LT, Engineer C, Kim TH (2014). Regulation of drought tolerance by the F-box protein MAX2 in Arabidopsis(1[C][W][OPEN]). Plant Physiol.

