# Supplementary material for: Identification and characterization of GmMYB118 responses to drought and salt stress
Source: BMC Plant Biol. 2018 Dec 3;18:320. doi: 10.1186/s12870-018-1551-7 (PMC6276260; doi:10.1186/s12870-018-1551-7)
Supplement: Supplementary file 1 — For disinfection of Arabidopsis thaliana seeds, the seeds of WT and EX (independent transgenic lines 4, 5 and 6) lines was disinfected with sodium hypochlorite. After 3 days of vernalization at 4 °C, the plates containing the seeds was transferred to a growth chamber. For statistical methods of data, the data shown are the means ± SDs (n = 80) of three experiments. ANOVA tests demonstrated that there were significant differences (∗ P < 0.05, ∗∗ P < 0.01). Expression of AtActin was analyzed as a loading control in Arabidopsis (Table S1). Expression of GmActin was analyzed as a control in soybean (Table S1.) Figure S1. Quantitative gene expression of all MYB-related TF family members in soybean. The tissue expression data of quantified prediction for a diverse set of fourteen tissue types from the soybase website (http://soybase.org/soyseq/). Figure S2. Subcellular localization and expression level of GmMYB118. GmMYB118 was localized in the nucleus in Arabidopsis mesophyll protoplasts (A). Scale bars = 20 μm. GmMYB118 gene expression in ectopic expression and WT (Col-0) plants (B). Figure S3. Germination rate of OE lines under PEG treatment. Images of germinating EX and WT (Col-0) seeds after 72 h under 0, 3, 6 and 9% PEG6000 treatment (A). The germination of the WT and EX plants of sown on 1/2-strength MS growth media with 0% (B), 3% (C), 6% (D) and 9% PEG6000 (E) were monitored until to 72 h. Figure S4. Germination rate of EX lines under NaCl treatment. Images of germinating EX and WT (Col-0) seeds after 72 h under 0, 75, 100 and 125 mM NaCl treatment (A). The germination of the WT and EX plants of sown on 1/2-strength MS growth media with 0 (B), 75 (C), 100 (D) and 125 mM NaCl (E) were monitored until to 72 h. Figure S5. Targeted mutagenesis in soybean hairy roots and GUS staining. The GUS staining of transgenic hairy roots revealed a transformation efficiency of approximately 50% (A). The target gene was amplified with specific primers and sequenced (AuGCT, China) [file 12870_2018_1551_MOESM1_ESM.pdf]

Supplementary figures and tables

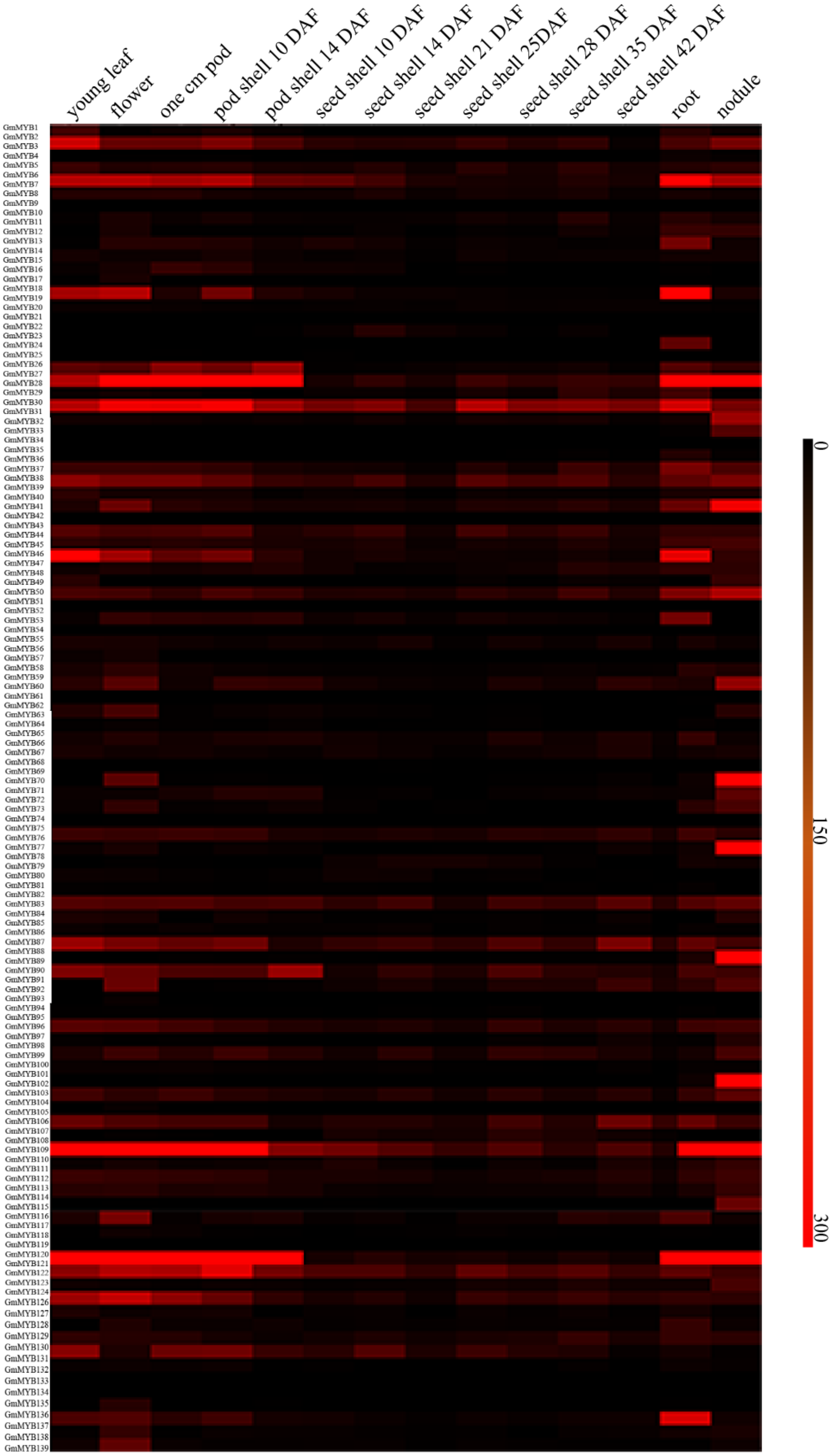

Figure S1

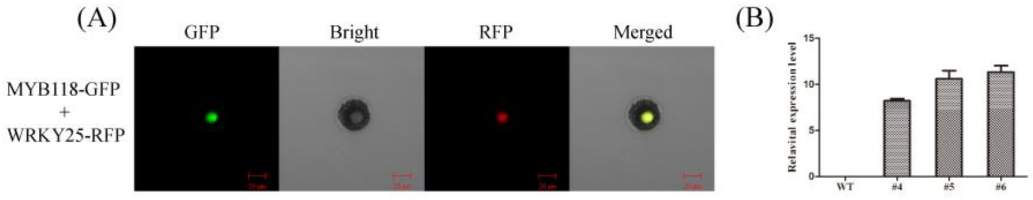

Figure S2

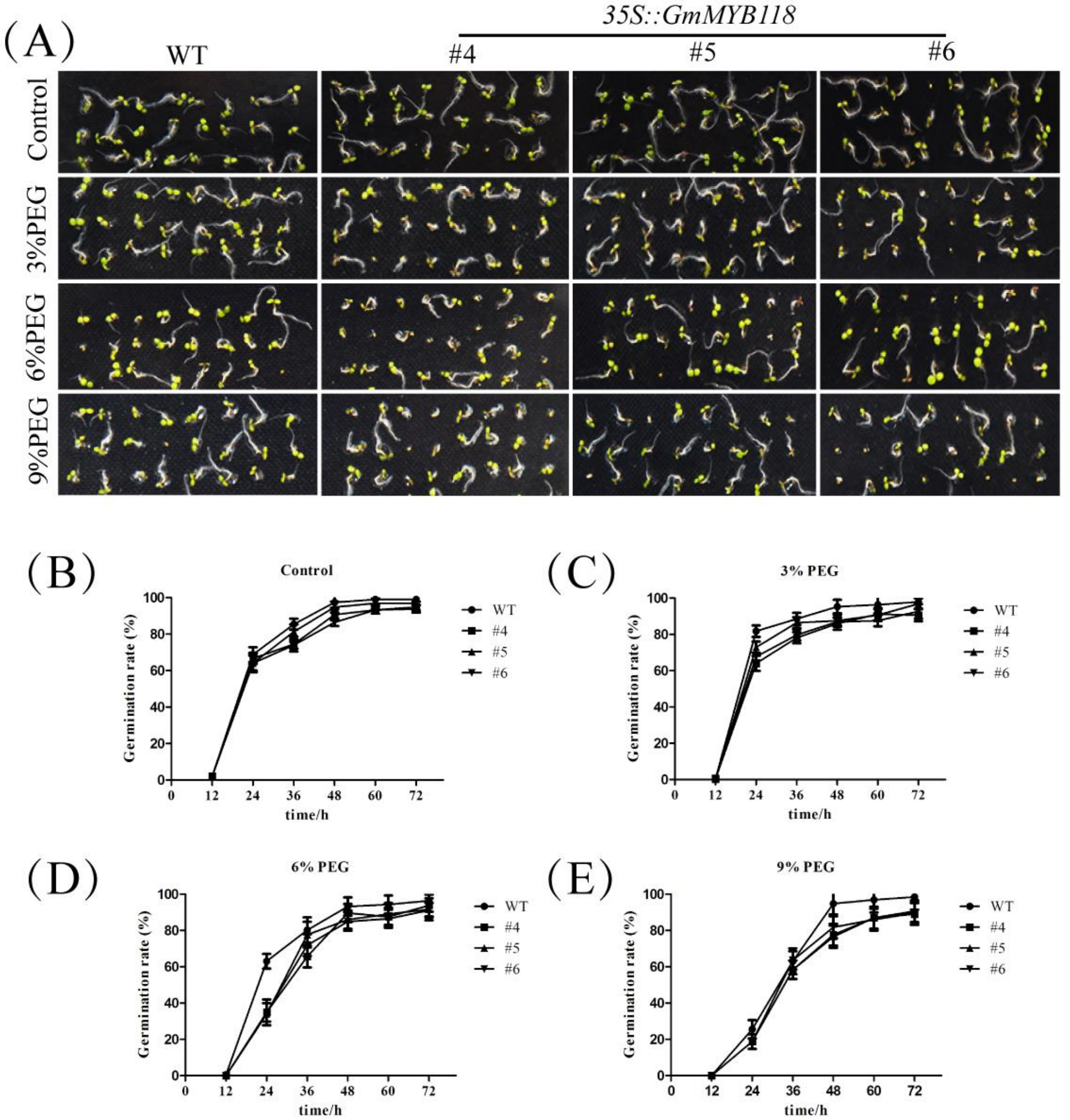

Figure S3

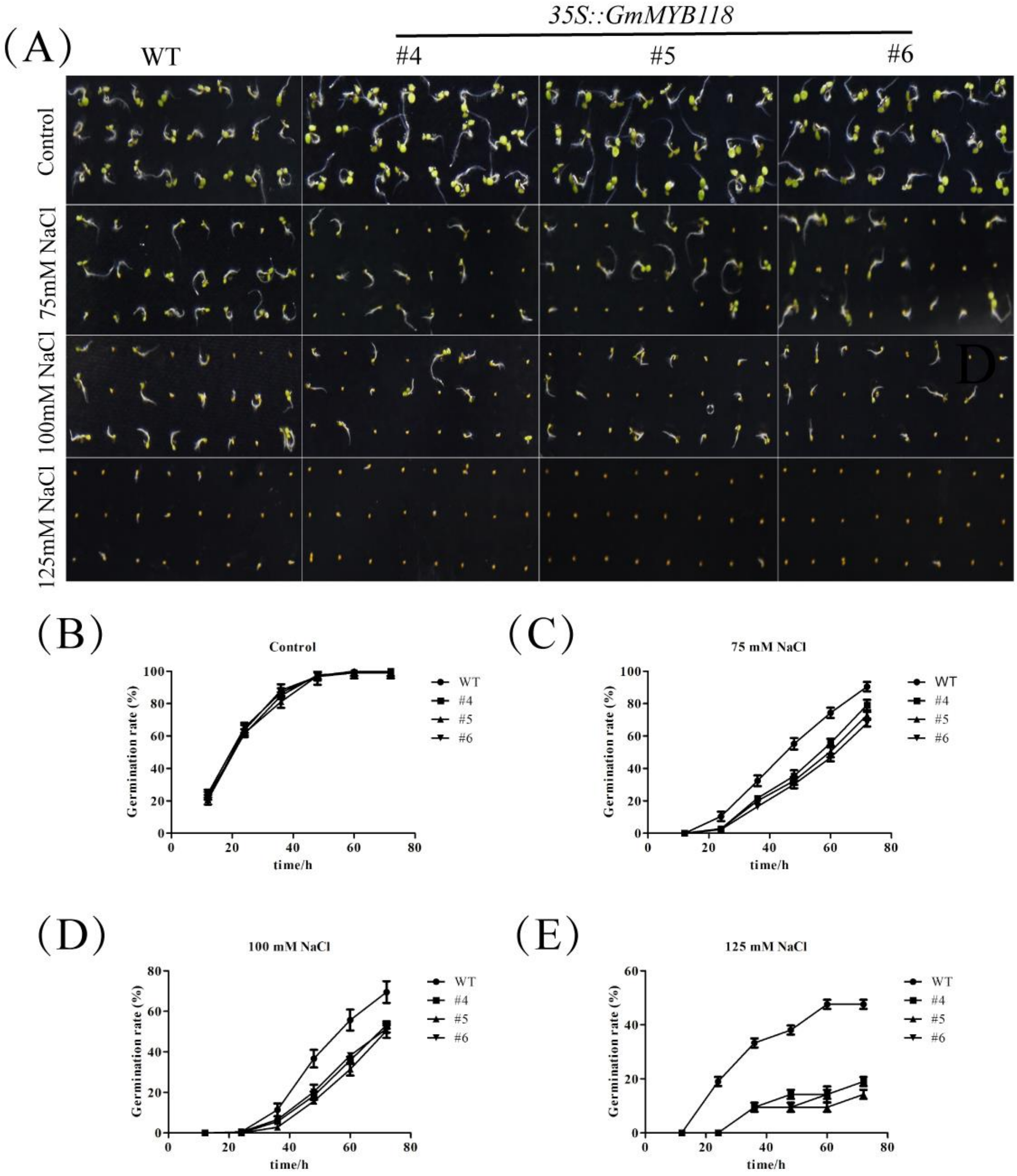

Figure S4

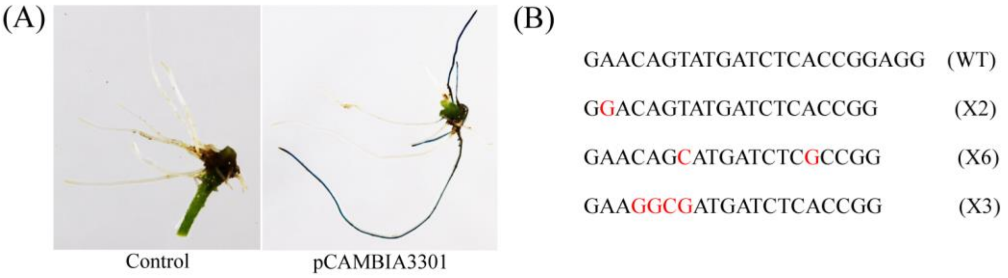

Figure S5

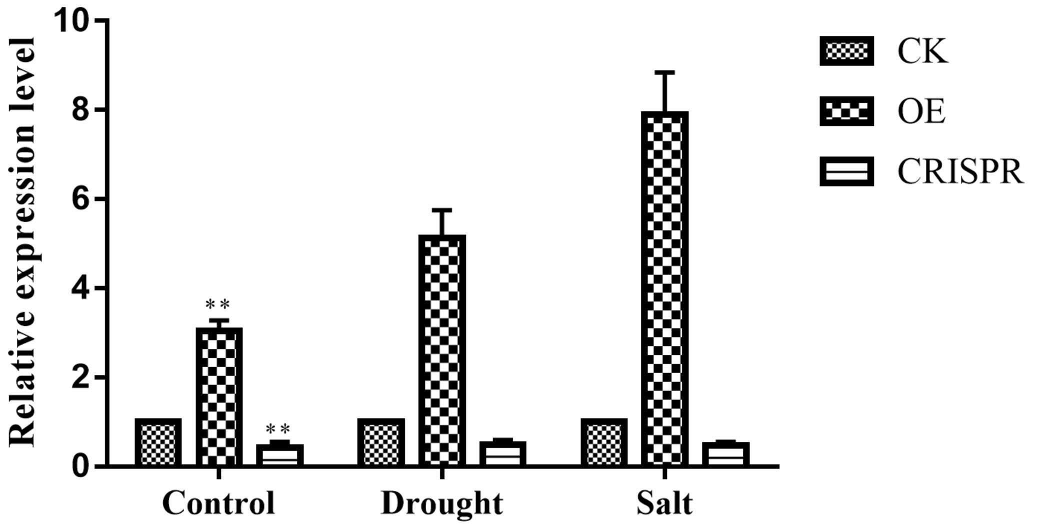

Figure S6

	Forward Primer	Reverse Primer
RT-AtNCED3	ATTGCTTCTGCTTCCATCTCT	TTCTGTTGACCATCCCTGCT
RT-AtRAB18	AGCTCGGAGGATGATGGACA	AGCCACCAGCATCATATCCG
RT-AtDREB2A	GGTTGGCCCAATGATGTGGA	CCATCCTTTCCCTCGAGCTG
RT-AtCOR47	AGGATTCACCAGCTGTCACG	AGGATTCACCAGCTGTCACG
RT-AtKIN1	TTAACACCTCCCACTGCCG	TTGTTCAGGCCGGTCTTGTC
RT-AtKIN2	CAAGAATGCCTTCCAAGCC	TTAACACCTCCCACTGCCG
RT-AtCOR15	GCGATGTCTTTCTCAGGAGC	CGAACTGAGTTTTCTGGCCG
RT-AtADH1	CTCCACCGCAGAAACACGA	TCCAACACTCTCAACAATCCC
RT-AtActin	AAGTCTTGTTCCAGCCCTCG	AAGTCTTGTTCCAGCCCTCG
RT-GmActin	ACATTGTTCTTAGTGGTGGCT	CTGTTGGAAGGTGCTGAG
RT- GmMYB7	AGAACAAGTTCCGCAAGA	AACGGCAAAGGCTCAATG
RT- AtMYB20	AACATTACATAGCGAAGC	ACCGAAGGTATAAACTCC
RT- AtMYB31	CGACGAAGCAACCTCAAT	AAGAACCAACCCCAAACT
RT- AtMYB49	AGCCAATAGGAGGGGTTA	TGTTGTGTGAAGGGAGGA
RT- AtMYB75	CAACAAGAAATCCGAAGG	GGAGGTAATTAACCCACC
RT- AtMYB81	TTGGTGTATTTTGTTGGC	GTGTTGTTCTTGGGGTGT
RT- AtMYB92	GAGGTGATAATGGTGTGA	ACTGGCTGAAGAGTAGTG
RT- AtMYB105	TTGGTGTTCCTTTGTTGGC	TCCTCGCTTTTTGTCCTG
RT- AtMYB110	AGCCAATAGGAGGGGTTA	TGTGTTGTGTGAAGGGAG
RT- AtMYB118	ATCATACTGTTCGGAGTCAG	CAGACACTGTAGAGACCTTGTT

Table S1

	Forward Primer	Reverse Primer
MYB118-1302	GGGACTCTTGACCATGATGTCTCGCGCCTCCT	TCAGATCTACCATGGCAGCAACACTAATGATGCTTTCT
MYB118-3301	GGACTCTTGACCATGATGTCTCGCGCCTCCT	ATTCGAGCTGGTCACCAGCAACACTAATGATGCTTTCT
MYB118-GFP	TATCTCTAGAGGATCCATGTCTCGCGCCTCCT	TGCTCACCATGGATCCAGCAACACTAATGATGCTTTCT
WRKY25-RFP	CCCAGATCACTAGTATGTCTTCCACTTCT	CTTGCTCACCATGGATCCTCACGAGCGTAGC
sgRNA seeds	ATTGGAACAGTATGATCTCACCGG	AAACCCGGTGAGATCATACTGTTC
U6-sgRNA	GCAGTATGCAGAGCAGAGTCA	CCGGTGAGATCATACTGTTC
pCas9	TGTAAAACGACGGCCAGT	CAGGAAACAGCTATGACC

Table S2
